# Divergent sorting of a balanced ancestral polymorphism underlies the establishment of gene-flow barriers in *Capsella*

**DOI:** 10.1038/ncomms8960

**Published:** 2015-08-13

**Authors:** Adrien Sicard, Christian Kappel, Emily B. Josephs, Young Wha Lee, Cindy Marona, John R. Stinchcombe, Stephen I. Wright, Michael Lenhard

**Affiliations:** 1 Institut für Biochemie und Biologie, Universität Potsdam, Karl-Liebknecht-Strasse 24-25, Haus 26, 14476 Potsdam-Golm, Germany; 2 Department of Ecology & Evolutionary Biology, University of Toronto, 25 Willcocks Street, Toronto, Ontario, Canada M5S 3B2

## Abstract

In the Bateson–Dobzhansky–Muller model of genetic incompatibilities post-zygotic gene-flow barriers arise by fixation of novel alleles at interacting loci in separated populations. Many such incompatibilities are polymorphic in plants, implying an important role for genetic drift or balancing selection in their origin and evolution. Here we show that *NPR1* and *RPP5* loci cause a genetic incompatibility between the incipient species *Capsella grandiflora* and *C. rubella*, and the more distantly related *C. rubella* and *C. orientalis*. The incompatible *RPP5* allele results from a mutation in *C. rubella*, while the incompatible *NPR1* allele is frequent in the ancestral *C. grandiflora*. Compatible and incompatible *NPR1* haplotypes are maintained by balancing selection in *C. grandiflora*, and were divergently sorted into the derived *C. rubella* and *C. orientalis*. Thus, by maintaining differentiated alleles at high frequencies, balancing selection on ancestral polymorphisms can facilitate establishing gene-flow barriers between derived populations through lineage sorting of the alternative alleles.

## Abstract (web summary)

A hybrid incompatibility between *Capsella* plant species is due to an interaction between two immune regulators. Here, the authors show that highly divergent haplotypes result from balancing selection in the ancestral lineage and their sorting into derived lineages facilitated the evolution of the incompatibility.

## Graphical abstract

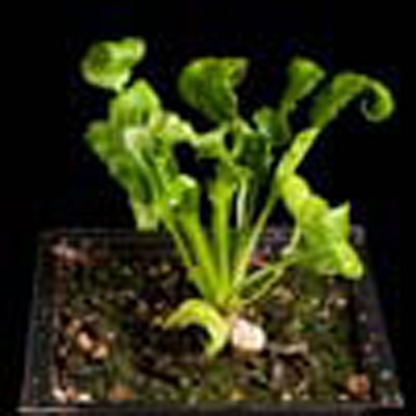

Speciation proceeds by the establishment of barriers to gene-flow between different populations, ultimately resulting in reproductive isolation^1^. Gene-flow barriers can act at the prezygotic stage, for example, by restricting the temporal overlap of the reproductive periods, or at the post-zygotic stage, by decreasing the fitness of hybrid progeny. Such post-zygotic barriers to gene flow often result from the establishment of Bateson–Dobzhansky–Muller incompatibilities (BDMIs)^1^,^2^,^3^,^4^. According to this model, novel alleles at one or more loci become fixed in separated populations, either due to genetic drift or selection, and produce detrimental consequences when combined in hybrid offspring after crossing^2^. Several loci causing BDMIs have been isolated from plant and animal species^3^,^5^. In plants, an autoimmune response termed hybrid necrosis represents a prominent manifestation of BDMIs^6^,^7^,^8^,^9^,^10^,^11^,^12^,^13^. Hybrid-necrosis results from the constitutive activation of the plant immune response due to a mismatch between different allelic versions of proteins involved in the response to pathogen infection. In particular, a comprehensive analysis of hybrid-necrosis cases within *Arabidopsis thaliana* has highlighted the family of nucleotide-binding leucine-rich repeat (NB-LRR) proteins as particularly prone to giving rise to hybrid necrosis^14^. NB-LRR proteins activate immune responses after detecting specific pathogen-derived proteins or their effects on host proteins and are highly variable between different genotypes^15^,^16^. Despite this progress in identifying causal BDMI genes, the evolutionary history and potential selection acting on incompatible alleles remain incompletely understood. In particular, the observation that in contrast to animals, many BDMIs are polymorphic within plant species, with segregating compatible and incompatible alleles at the causal loci, suggests a greater role for either genetic drift and/or balancing selection in the origin of BDMIs in plants than in animals^4^,^5^; however, the relative role of drift versus balancing selection remains uncertain.

One scenario where polymorphic alleles may make an important contribution to the establishment of gene-flow barriers is speciation associated with the transition from outbreeding to selfing, which has occurred many times independently in flowering plants^17^. Transitions to selfing entail a loss of heterozygosity, low effective population sizes and often genetic bottlenecks in the selfing populations. This can lead to a loss of both neutral and selected polymorphisms, thereby converting standing variation into fixed differences between different selfing populations^17^. Once fixed for alternative, functionally differentiated alleles, only one novel incompatible mutation to an interacting locus would be required to establish a BDMI between the derived populations^5^. This scenario could explain the observation that the transition to selfing appears to accelerate the establishment of post-zygotic gene-flow barriers between different selfing lineages and with the ancestral outbreeding species^17^,^18^,^19^,^20^.

The genus *Capsella* provides a tractable experimental system for testing competing models of the evolution of BDMIs associated with the transition to selfing. Within the last 100,000 years, the predominantly self-fertilizing *C. rubella* evolved from the outbreeding ancestor *C. grandiflora* via break-down of self-incompatibility and an associated severe genetic bottleneck^21^,^22^,^23^. An earlier speciation event ∼840,000 years ago^24^ gave rise to *C. orientalis*, which experienced an independent transition to selfing, from a presumed *C. grandiflora*-like ancestor^25^. While *C. grandiflora* is largely restricted to Greece, *C. rubella* is found around the Mediterranean and beyond, and *C. orientalis* occurs in Central Asia^25^. *C. grandiflora* and *C. rubella* co-occur in Greece, with more recent gene flow between them^26^. Here we exploit this system to show conclusively that a gene underlying a polymorphic BDMI is maintained as polymorphic by balancing selection in the ancestral outcrosser *C. grandiflora*. Independent transitions to selfing have led to the fixation of alternative alleles in the derived selfers *C. rubella* and *C. orientalis*, contributing to a genetic incompatibility between them.

## Results

### A two-locus, immune-related incompatibility in *Capsella*

**Figure 1:**
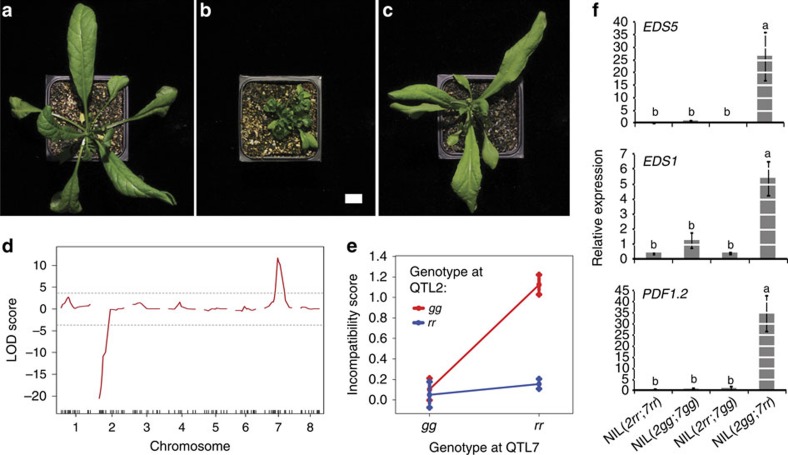
Incompatibility between *C. grandiflora* and *C. rubella* due to a constitutive immune response. (**a**) *C. rubella*, (**b**) incompatible *C. grandiflora* × *C. rubella* RIL hybrid and (**c**) *C. grandiflora* plant. Scale bar, 1 cm. (**d**) QTL mapping for stunted growth in the RIL population (*n*=142). LOD scores are multiplied by the sign of the additive effect of the locus. Dashed line represents the genome-wide 5% significance threshold. (**e**) Interaction plot of *grandiflora* (g) and *rubella* (r) alleles at QTL2 and QTL7. Incompatibility scores were determined based on visible inspection of phenotypes. Values correspond to the means±s.e.m for *n*=21, 18, 9 and 78 for the genotype groups (2*gg;7gg),* (2*rr; 7gg), (*2*gg; 7rr*) and (2*rr; 7rr)*, respectively. (**f**) Expression of immune-response genes determined by quantitative reverse transcription–PCR normalized to *Capsella TUB6*. Mean±s.e.m. of three biological replicates is shown. Letters indicate significant differences as determined by Tukey’s honest significant difference test (*α*=0.05). Means belonging to the same group, as indicated by the same letters, are not significantly different.

To determine whether genetic incompatibilities exist between the recently diverged *Capsella* species, we analysed a *C. grandifora* × *C. rubella* recombinant inbred line (RIL) population^27^ for incompatible phenotypes. In 13.3% of the RILs (19 of 142), we observed strongly retarded growth and severe stunting (Fig. 1a–c, Supplementary Table 1). Quantitative trait locus (QTL) mapping identified two major QTL on chromosomes 2 and 7 as responsible for the growth defect (Fig. 1d, Supplementary Fig. 1a). Plants homozygous for the *grandiflora* allele at QTL2 (*2gg*) and for the *rubella* allele at QTL7 (*7rr*) display a reduced biomass, indicating a strong negative epistatic interaction between these loci (Fig. 1e, Supplementary Fig. 1b). To validate the QTL mapping results, we generated near-isogenic lines (NILs) segregating for the two QTL regions in an otherwise largely *C. rubella*-derived background (Supplementary Table 2). Analysis of these NILs confirmed the incompatibility in 2*gg; 7rr* plants (Supplementary Fig. 1b). While the incompatible *grandiflora* QTL2 allele was fully recessive, the *rubella* QTL7 allele was semi-dominant (Supplementary Fig. 1b). We focussed our subsequent analysis on these NILs, as no suitable inbred lines are available for the obligate outbreeder *C. grandiflora*; as a result, genetic background variation between *C. grandiflora* individuals could confound any comparisons between the parental species and the NILs.

At the cellular level, reduced leaf growth in affected hybrids was largely caused by impaired cell expansion (Supplementary Fig. 1c). Several similar genetic incompatibilities segregating within *A. thaliana* result from an ectopic autoimmune response including necrosis^8^,^13^. While the incompatible *Capsella* hybrids did not show any ectopic cell death (Supplementary Fig. 2b), expression of ENHANCED DISEASE SUSCEPTIBILITY 1 (*EDS1*) and *EDS5* is strongly upregulated in incompatible hybrids (Fig. 1f); both genes are required for the accumulation of the plant hormone salicylic acid and establishment of systemic acquired resistance in a feed-forward loop^28^,^29^,^30^. Expression of the jasmonate- and ethylene-responsive gene PLANT DEFENSIN 1.2 (ref. 31) was also upregulated, in contrast to several other markers of an activated pathogen response (Supplementary Fig. 2a). As in *A. thaliana*^13^, the stunted growth resulting from this autoimmune response could be suppressed by growth at high temperature (Supplementary Fig. 2c). Thus, a two-locus, negative epistatic interaction causes symptoms resembling a constitutive activation of the immune response and stunted growth in *C. grandiflora* × *C. rubella* hybrids.

### Identification of incompatibility loci

**Figure 2:**
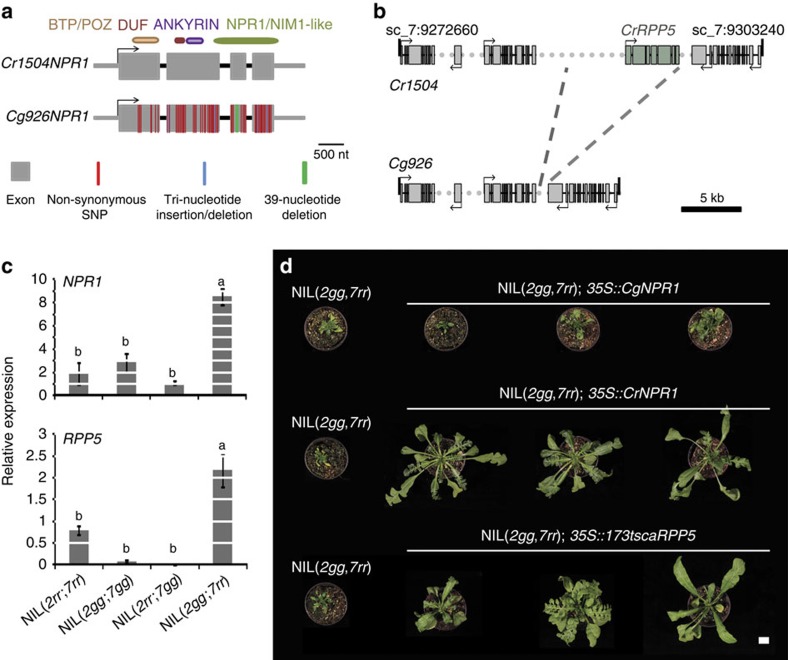
Genetic basis of the incompatibility between *C. grandiflora* and *C. rubella*. (**a**) Gene structure, location of functional domains and polymorphisms between *C. grandiflora* and *C. rubella NPR1* alleles from the RIL population. Rectangles are coding sequence, lines are untranslated regions and introns. (**b**) Gene structure and genomic organization of *RPP5* region from the RIL parents. Rectangles and solid lines are exons and introns; dashed line represents intergenic sequence. (**c**) Expression of *NPR1* and *RPP5* determined by quantitative reverse transcription–PCR normalized to *Capsella TUB6*. Mean±s.e.m. of three biological replicates is shown. Letters indicate significant differences as determined by Tukey’s honest significant difference test (*α*=0.05). Means belonging to the same group, as indicated by the same letters, are not significantly different. (**d**) Rescue of incompatible phenotype by suppression of *RPP5* expression (bottom) and by transgenic expression of the *CrNPR1* (middle), but not of the *CgNPR1* allele (top). Scale bar, 1 cm.

Fine mapping in the segregating NIL progeny localized the causal gene underlying QTL2 to a 40-kb interval containing the *Capsella* orthologue of the immune-response factor NON-EXPRESSOR OF PR-GENES1 (*NPR1*; ref. 32) (Fig. 2a; Supplementary Fig. 3a; Supplementary Table 3). *NPR1* is a central regulator of systemic acquired resistance, a systemic response to pathogen infection that does not induce cell death, but leads to an increase in salicylic acid content, which in turn upregulates *EDS1* and *EDS5* expression^28^,^29^,^30^, similar to the incompatible phenotype in *Capsella*. Expression analysis indicated that *NPR1* was not robustly differentially expressed between NIL(*2rr; 7rr*) and NIL(*2gg; 7gg*) plants (Fig. 2c), suggesting that the incompatibility is due to differences in the coding sequence. To test this, incompatible NIL(*2gg; 7rr*) plants were transformed with constructs for constitutive expression of the *C. rubella NPR1* complementary DNA (cDNA; *35S::CrNPR1*) or the *C. grandiflora NPR1* cDNA (*35S::CgNPR1*). While the latter had little effect on the morphological phenotype despite very high expression levels, expression of *35S::CrNPR1* abolished the stunted growth of NIL(*2gg; 7rr*) plants (Fig. 2d; Supplementary Fig. 3c,d). At the gene-expression level, *35S::CrNPR1* transgenic plants showed a strong reduction in expression of the marker genes *EDS1*, *EDS5* and *PDF1.2* (Supplementary Fig. 3i–k); expression of these markers was also reduced in *35S::CgNPR1* transgenic plants, albeit to a lesser extent for *EDS1* and *EDS5*, suggesting that the very high-level expression of *CgNPR1* in the two assayed lines (2 and 3 in Supplementary Fig. 3c–k) can partially uncouple the enhanced defence-gene expression from the stunted growth in the incompatible hybrids. We conclude that the *NPR1* gene represents the incompatibility locus on chromosome 2, and the causal difference(s) between the two alleles appears to affect the encoded proteins. The two alleles show a high level of diversity within the *NPR1* coding sequence with 77 non-synonymous substitutions, 3 single-codon insertions/deletions and a larger deletion of 39 nucleotides in the *grandiflora* allele (Fig. 2a; Supplementary Fig. 4b).

The causal gene underlying QTL7 was mapped to a 120-kb interval that contains the closest *Capsella* homologue to the TIR-NB-LRR pathogen-response regulators RECOGNITION OF PERONOSPORA PARASITICA 5 (*RPP5*; ref. 33) and BAL/SUPPRESSOR OF NPR1-1,CONSTITUTIVE1 (*BAL/SNC1*; refs 34, 35) (Fig. 2b; Supplementary Fig. 3a; Supplementary Table 3). While the gene is present in the *C. rubella* allele, it is deleted from the specific *C. grandiflora* allele segregating in the RIL population (Fig. 2b). To test the role of *CrRPP5* in causing the incompatible phenotype, its expression was reduced in incompatible NIL(*2gg; 7rr*) plants by specific miRNA-induced gene silencing^36^; this was sufficient to suppress their growth defect and the constitutive defence-gene expression (Fig. 2d; Supplementary Fig. 3c,e–k). Thus, an incompatibility between the *grandiflora NPR1* and the *rubella RPP5* alleles underlies the constitutive immune response and growth retardation in the affected hybrids. Both *NPR1* and *RPP5* showed higher expression levels in incompatible NIL plants than in compatible genotypes (Fig. 2c); for *NPR1* and to a lesser extent for *RPP5*, this enhanced expression was suppressed in the rescued *35S::CrNPR1* and *RPP5* knock-down transformants (Supplementary Fig. 3g,h), suggesting that positive feed-back from the constitutive immune response is responsible for their elevated expression in the incompatible NIL plants. As *RPP5* homologues in *A. thaliana* have been identified as strongly overrepresented among loci causing hybrid necrosis^14^, our findings suggest that their propensity for giving rise to incompatible alleles is an evolutionarily more wide-spread phenomenon that can give rise to between-species incompatibilities.

### Evolutionary history of the incompatible alleles

**Figure 3:**
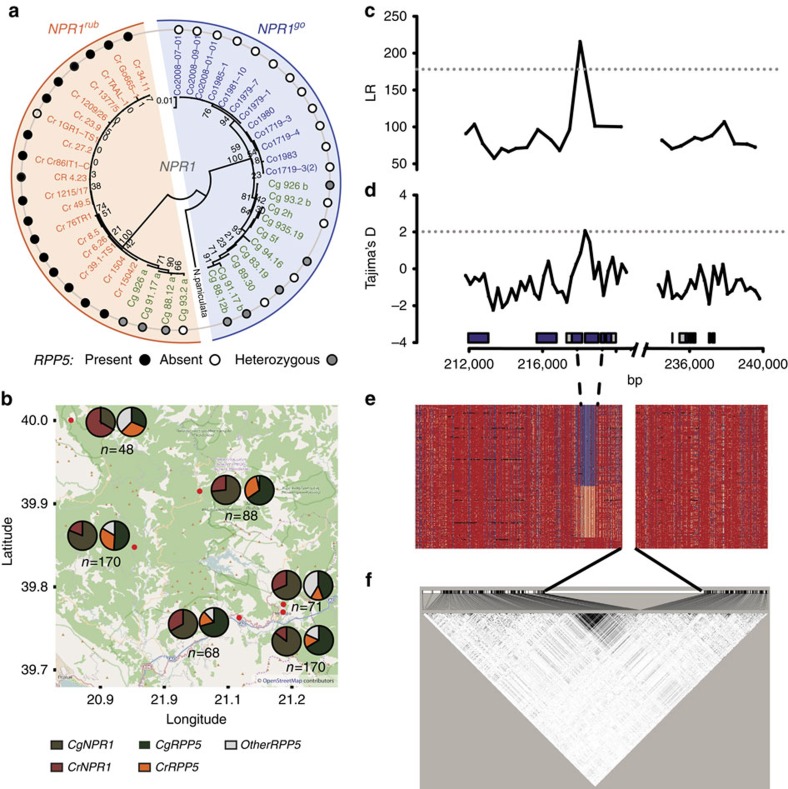
Balancing selection maintains functionally divergent haplotypes at *NPR1*. (**a**) Phylogeny of *NPR1* alleles based on a 700-bp fragment from *C. grandiflora* (green), *C. rubella* (orange) and *C. orientalis* accessions (blue). Different *NPR1* alleles from the same heterozygous *C. grandiflora* accession are indicated by ‘a’ and ‘b’ after accession name. The incompatible *NPR1* allele from the *Cg926* parent of the RIL population is termed ‘926b’. Presence or absence of *RPP5* is indicated. (**b**) Allele frequency distribution at the *NPR1* and *RPP5* loci within natural *C. grandiflora* populations. *CrRPP5* and *CgRPP5* indicate the frequency of the *Cr1504 RPP5* and the *Cg926 RPP5* alleles, respectively. ‘Other *RPP5*’ indicates the frequency of individuals, where none of these two alleles could be amplified. The sample size for each population is indicated on the figure under each pie chart (*n*). The map is based on OpenStreetmap (https://www.openstreetmap.org/copyright). (**c**,**d**) Composite likelihood ratio (LR) test of selection in 50-SNP windows (**d**) and estimates of Tajima’s D in 500 bp windows with 250 bp overlaps (**d**) from 178 resequenced *C. grandiflora* individuals. Dashed line shows the 99.9% percentile across chromosome 2 as the genomic control. (**e**) Haplotype structure in the region surrounding *NPR1* in the 178 *C. grandiflora* individuals. For each called variant, red indicates the sample is homozygous for the *C. rubella* reference allele, pink is heterozygous and blue is homozygous for the alternate allele. (**f**) Pairwise linkage disequilibrium between variants across the region. Black: r^2^=1, white: r^2^=0. Gaps in **c**–**f** are due to missing data after filtering in a 13.7-kb interval.

To gain insights into the evolutionary history of the incompatible alleles, we sequenced an ∼700-bp fragment surrounding the 39-nt deletion in *NPR1* from 10 *grandiflora*, 18 *rubella* and 10 *orientalis* accessions. The resulting gene phylogeny was split into two distinct clades almost as distant from each other as from the outgroup *Neslia paniculata NPR1* sequence^37^ (Fig. 3a; Supplementary Fig. 5a). One clade contained all of the *C. rubella* and some of the *C. grandiflora* alleles (termed *NPR1*^*rub*^), while the other contained the remaining *C. grandiflora* alleles, including the incompatible one from our RIL population (allele Cg926b in Fig. 3a), and all *C. orientalis* alleles (termed *NPR1*^*go*^). While overall *NPR1*^*go*^ alleles are somewhat closer to other *Brassicaceae* sequences than *NPR1*^*rub*^ alleles, all *NPR1*^*go*^ alleles contain the derived 39-nt deletion not found in other *Brassicaceae* species (Supplementary Fig. 4a,b).

*RPP5* was present in 17 out of 18 *rubella* accessions and absent in all of the 10 *orientalis* accessions (Fig. 3a). In *grandiflora*, *RPP5-*presence and -absence alleles segregate irrespective of the *NPR1* allele found in the accessions. The different *RPP*5 haplotypes seen in *C. rubella* reflect a subset of the haplotype variation in the ancestral *C. grandiflora* (Supplementary Fig. 5b), and the *RPP5* deletion in *C. grandiflora/orientalis* is evolutionarily derived, as *RPP5* homologues are found in the syntenic position in all other analysed *Brassicaceae* genomes except for two of the three subgenomes of *Brassica rapa* (Supplementary Fig. 5c). Thus, the distribution of different *NPR1* and *RPP5* haplotypes indicates that at both loci ancestral polymorphisms were present before the divergence of the Western *Capsella* lineage, including *C. grandiflora* and *C. rubella*, and the Eastern lineage, including *C. orientalis* (ref. 25).

To determine the geographical distribution of the different *NPR1* and *RPP5* alleles within the outbreeding *C. grandiflora*, we genotyped six natural *C. grandiflora* populations from the Zagori Mountains in Greece. All six populations segregated for the *NPR1*^*rub*^ and *NPR1*^*go*^ alleles, as well as the presence or absence of *RPP5* at high frequencies (Fig. 3b). We asked whether segregation of different alleles at both loci within *C. grandiflora* was associated with the occurrence of the incompatible phenotype. However, neither progenies of plants collected from the six above populations from the wild (*n*=749 progeny plants) nor offspring of controlled crosses between *C. grandiflora* plants heterozygous at *NPR1* and for presence/absence of *RPP5* (*n*=504 progeny plants) showed a robust incompatible phenotype associated with the expected incompatible genotype.

This absence of incompatible phenotypes despite the segregation of different alleles at both loci could reflect the activity of modifier alleles at other loci that would have been lost in *C. rubella* or allelic heterogeneity, with only some *NPR1*^*go*^ alleles or some *RPP5* alleles causing incompatibility. To test for allelic heterogeneity, we crossed different *C. rubella* and *C. grandiflora* accessions and analysed the progenies (Supplementary Fig. 6 and 7a,b; Supplementary Discussion). An incompatible phenotype associated with the *2gg; 7rr* genotype was observed when combining the *RPP5* allele from *Cr1504* (the *C. rubella* parent of the RIL population) with any *NPR1*^*go*^ alleles; however, this was not seen for any other *RPP5* alleles, including one from the same haplotype group as that in *Cr1504* (in *Cr22.5*, cross 21; Supplementary Fig. 6). This suggests that a novel mutation(s) in *RPP5* in the *Cr1504* lineage led to the incompatible allele and explains the absence of incompatible phenotypes in *C. grandiflora*. In three of the crosses involving *C. rubella* accessions other than *Cr1504* (crosses 3, 9 and 10), we observed similar incompatible phenotypes to the one above, with stunted growth, increased expression of immune-response genes and suppression by high temperature (Supplementary Figs 7b and 8a,b). Importantly, however, these incompatibilities do not involve the *RPP5* locus, but do show tight linkage to *NPR1* in at least two cases (crosses 3 and 9; Supplementary Fig. 7b; Supplementary Discussion). Thus, mutations to more than just one interacting locus can lead to the evolution of genetic incompatibilities with one of the *NPR1* haplotype groups in *C. grandiflora*, suggesting that the presence of highly divergent alleles in a population can facilitate the establishment of BDMIs.

### Balancing selection maintains divergent *NPR1* haplotypes

The above observations raise the question how the *NPR1* polymorphism is maintained in *C. grandiflora*? To address this, we analysed the *NPR1* region from 178 resequenced *C. grandiflora* individuals from a natural population in Northern Greece^26^. This demonstrated a strong signal of balancing selection on *NPR1*, with a highly significant positive peak in Tajima’s D (that is, an excess of intermediate frequency alleles) and an extreme composite likelihood ratio test score (Fig. 3c,d). Since both of these tests are conducted in the context of the genome-wide empirical distribution, these extreme values are unlikely to be explained by demographic effects, and instead are indicative of balancing selection. The *NPR1* locus also shows an unusual haplotype structure with strong linkage disequilibrium over 85% of the coding-sequence length (positions 217,869 to 219,840 on scaffold 2; *NPR1* coding sequence is from position 217,594 to 219,740) (Fig. 3a,e,f). This unusual haplotype structure could be due to a partial selective sweep or long-term balancing selection with recombination suppression; the latter explanation is most likely, as the haplotype diversity appears to be ancient (fourfold diversity between haplotypes is 0.115, roughly six times higher than the average genome-wide pairwise diversity^21^,^22^,^23^). Thus, the strong differentiation between the two *NPR1* haplotype groups seen in the phylogenetic tree based on the 700-bp fragment reflects the long-term maintenance of highly differentiated *NPR1* haplotypes in the ancestral *C. grandiflora* population through balancing selection and their subsequent sorting into the two selfing species (Fig. 3a). A comparable analysis for *RPP5* was not possible, as strong structural heterogeneity of the locus precluded precise genotype reconstruction from next-generation sequencing data. Thus, we conclude that balancing selection maintains two strongly divergent *NPR1* haplotype groups.

We next asked whether balancing selection on *NPR1* could be due to different activities of the two allele clades in setting the basal level of immune-system activation. To test this possibility, we analysed transcriptome data for 99 *C. grandiflora* individuals from the same sample as above^38^. No single gene showed a statistically significant expression difference between the three *NPR1* genotype groups, likely reflecting the limited statistical power of the experiment. We therefore examined overrepresented functional categories among the 1,000 genes with the lowest *P* values when testing for an association of gene expression and *NPR1* genotype. This identified a significant overrepresentation of genes involved in pathogen response, both using MapMan analysis^39^ and MASTA-based comparisons^40^ with published *A. thaliana* microarray data (Supplementary Fig. 9); in particular, genes upregulated after pathogen- or elicitor-treatment in *A. thaliana* tend to be more strongly expressed in *C. grandiflora* plants homozygous for *NPR1*^*rub*^ alleles than in plants homozygous for *NPR1*^*go*^ alleles and *vice versa*. This suggests a functional difference between the two allele clades in basal immune-system activation as a reason for their maintenance by balancing selection.

### Genetic incompatibility between *C. rubella* and *C. orientalis*

**Figure 4:**
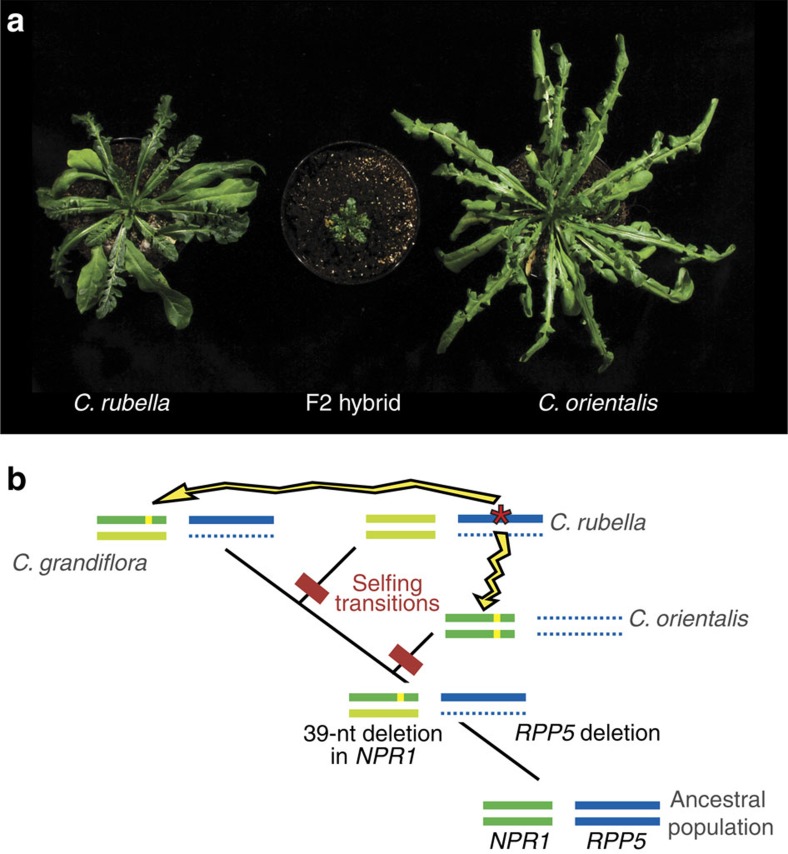
Divergent lineage sorting of ancestral polymorphisms. (**a**) Plants of *C. rubella 1504*, *C. orientalis 1983* and incompatible F2 hybrid. (**b**) Proposed evolutionary history of *NPR1* and *RPP5* loci in the genus *Capsella*. In an ancestral population before the divergence of the three species, a deletion of the *RPP5* locus occurred; in addition, the 39-nt deletion occurred in *NPR1* (yellow box in dark green *NPR1* allele) and the two *NPR1* haplotype groups diverged (light and dark green *NPR1* alleles). Following the independent transitions to selfing in *C. orientalis* and *C. rubella*, the *NPR1* haplotypes were divergently sorted; *C. orientalis* also became fixed for the *RPP5* deletion. In *C. rubella*, the incompatibility-inducing mutation in *RPP5* arose (red asterisks), leading to BDMIs with *C. grandiflora* and *C. orientalis* (yellow arrows).

The two selfing species *C. rubella* and *C. orientalis* were derived independently from a *C. grandiflora*-like ancestor and have fixed alternative ancestral *NPR1* alleles (Fig. 3a). In addition, *C. orientalis* appears to be fixed for the absence of *RPP5* (Fig. 3a; Supplementary Fig. 5). We therefore tested whether hybrids of *C. rubella Cr1504* and *C. orientalis* would show a similar incompatibility as above. F1 plants from a cross of *C. rubella Cr1504* to *C. orientalis* showed reduced growth, and this became more pronounced in the F2 (Fig. 4a; Supplementary Fig. 8c). Genotyping demonstrated that incompatibility is associated with the *NPR1*^*go*^ allele and *C. rubella RPP5* allele. In contrast to what was observed in *C. rubella* × *C. grandiflora* hybrids (Supplementary Fig. 1b), the incompatible alleles at both loci acted in a semi-dominant manner, thus establishing a robust BDMI (Supplementary Fig. 8d).

## Discussion

Our results identify a negative epistatic interaction between the *Capsella NPR1* and *RPP5* orthologues that causes a polymorphic BDMI between the selfing *C. rubella* and the ancestral outbreeder *C. grandiflora*, as well as between the two selfing lineages *C. rubella* and *C. orientalis*. We also show that the causal *NPR1* polymorphism is maintained in *C. grandiflora* by balancing selection, and the alternative alleles were divergently sorted into *C. rubella* and *C. orientalis* (Fig. 4b).

How could an incompatible interaction between *RPP5* and *NPR1* trigger a constitutive pathogen response? *NPR1* represents a central regulator of systemic acquired resistance; its salicylic acid and redox-dependent translocation into the nucleus after pathogen infection triggers large-scale transcriptional reprogramming, acting as a transcriptional co-regulator in concert with TGA-family transcription factors^30^. While overexpression of *BAL/SNC1* causes a constitutive immune response and stunted growth in otherwise wild-type *A. thaliana* plants^34^, a constitutively active mutation in *BAL/SNC1* was discovered as a suppressor of the *npr1* mutant phenotype, triggering a similar phenotype even in the absence of functional *NPR1* (ref. 35), and arguing for an *NPR1*-independent role of the *RPP5*-homologue *SNC1*. By contrast, work in *A. thaliana* has also demonstrated that *NPR1* function is required for race-specific downy-mildew resistance conferred by RPP5 and closely related TIR-NB-LRR proteins, highlighting a functional interaction between NPR1 and RPP5-like proteins^41^,^42^, as also suggested by our results in *Capsella*. However, the molecular details of this interaction have not been resolved. Our transcriptome analyses suggest that the compatible *C. rubella*-like *NPR1* allele is more active in promoting basal resistance-gene expression than the incompatible *C. grandiflora*-like allele; thus, it appears unlikely that the constitutive immune response is simply triggered by the additive effect of two somewhat more active signalling molecules, whose individual effects are insufficient to trigger the response. Although there is currently no molecular evidence for such an interaction, direct binding of the incompatible protein versions to trigger an immune response is conceivable.

What are the implications of our results for the evolution of BDMIs in plants? First, the compatible and incompatible *NPR1* alleles in *C. grandiflora* provide a clear example of how balancing selection as opposed to genetic drift can underlie the frequently observed intraspecific polymorphisms for BDMIs^4^,^5^. Second, the *Capsella* genus demonstrates how such balanced polymorphisms can contribute to BDMIs between independently derived selfing lineages (*C. rubella* and *C. orientalis*), when these become fixed for the alternative alleles, likely due to stronger genetic drift in the selfing lineages. Third, it is plausible to assume that such divergent lineage sorting of functionally differentiated alleles can accelerate the establishment of BDMIs when compared with a scenario without standing variation in the ancestral population. This is because after such divergent lineage sorting only one novel incompatible mutation would be required to an interacting locus, as was the case for *RPP5* in the *Cr1504* lineage. Furthermore, the results from additional *C. grandiflora* × *C. rubella* crosses suggest that the *NPR1* polymorphism underlies additional incompatibilities between *C. grandiflora* and *C. rubella* that involve at least one additional locus besides *RPP5*. In this view, balancing selection would facilitate the evolution of BDMIs via two effects. First, the long-term maintenance of the alternative alleles would allow their higher-than-average molecular divergence, increasing the risk of incompatibilities with novel alleles at interacting loci. In the case of *NPR1*, the extent of divergence between the *NPR1*^*rub*^ haplotypes in *C. rubella* and the *NPR1*^*go*^ haplotypes in *C. grandiflora* or *C. orientalis* is likely to be among the highest across the genome (*cf.*, Supplementary Fig. 5a); thus, mutations to genes that interact with *NPR1* are more likely to give rise to an incompatibility with one of the *NPR1* haplotypes than mutations at other loci that interact with genes with less divergent haplotypes. As a result, in the *C. rubella* lineage alleles that are incompatible with the *NPR1* haplotype not found in *C. rubella* (that is, *NPR1*^*go*^) should arise more frequently than alleles that are incompatible with the non-*C. rubella* alleles at other, less divergent loci. The second effect of balancing selection would be that the balanced frequencies of the alternative alleles make their divergent lineage sorting into derived populations more likely than for more strongly skewed allele frequencies. Consistent with this notion, we note that the absence/presence polymorphism for *RPP5* seen at high frequencies in *C. grandiflora* populations (Fig. 3a) appears to have undergone divergent lineage sorting into the two derived selfing species as well, with a deletion allele fixed in *C. orientalis*, and *RPP5* presence almost fixed in *C. rubella*. Whether the *RPP5* deletion seen in the *C. rubella* accession *Cr1GR1-TS1* (Fig. 3a) reflects incomplete lineage sorting, a novel deletion in the *C. rubella* lineage or post-divergence introgression of a deletion allele from *C. grandiflora* is currently unclear. More generally, incompatibilities originating from standing variation may be also more likely to arise from loci under balancing selection than loci subject to mutation-selection balance, since their historical maintenance at higher frequencies by selection (for example, frequency-dependent selection, spatially or temporally varying selection, genotype × environment interactions) implies that both alleles can be selectively favoured under certain conditions. In contrast, alleles under mutation-selection balance are likely to be at lower initial frequencies, deleterious, and selected against.

Even though it was not possible to resolve the different *RPP5* haplotypes in the resequenced *C. grandiflora* population, most likely due to substantial structural variation, we can assume that the same evolutionary forces act on this *R* gene as have been described for *R* genes in other plant species. *R* genes from natural populations generally show the highest levels of genetic variation in plant genomes and evidence of balancing or diversifying selection with maintenance of polymorphisms^15^,^43^. Thus, novel *R*-gene alleles appear to enter and rise to appreciable frequencies in populations more often than novel alleles at other, non-*R* genes; at the same time, they appear to be more likely to be maintained as polymorphisms, rather than becoming fixed in the populations^15^,^43^. In the case of *Capsella*, this should further promote the establishment of genetic incompatibilities involving *NPR1* and interacting *R*-genes, yet should delay or prevent fixation of incompatible *R*-gene alleles.

At present, it is unclear how large a contribution the studied BDMI between *NPR1* and *RPP5* has made to speciation in *Capsella*, given its largely recessive behaviour and polymorphism between *C. rubella* and *C. grandiflora*, and the biogeography and evolutionary history of the *C. orientalis* versus the *C. grandiflora*/*C. rubella* lineages^25^. However, despite this uncertainty it suggests a potentially more wide-spread importance for ancestral polymorphisms under balancing selection as a basis for the evolution of plant BDMIs, particularly when combined with divergent lineage sorting after a transition to selfing or other genetic bottlenecks. In fact, the evolution of male semi-sterility in hybrids of *japonica* and *indica* rice supports a broadly similar scenario^44^,^45^. The causal difference in one of the two interacting loci, a C-to-T change in *SaF*, is found as a trans-specific polymorphism in widely diverged rice species and segregates at intermediate frequencies in current populations of the wild rice *Oryza rufipogon*^44^,^45^. Though not formally demonstrated, this suggests that the two alleles have been maintained by long-term balancing selection^46^. A novel mutation to the interacting locus *SaM* occurred in a subpopulation of *O. rufipogon*, causing a BDMI in *SaF*^*−*^ *SaM*^*−*^/*SaF*^*+*^ *SaM*^*+*^ hybrids. During rice domestication, the *SaF*^*−*^ *SaM*^*−*^ haplotype was preferentially retained in *japonica* rice, while the *SaF*^*+*^ *SaM*^*+*^ haplotype is prevalent in *indica* rice, contributing to the hybrid sterility between the subspecies. Thus, while the relative timing of events seems to differ from the *Capsella* example, the evolution of *SaF/SaM*-based hybrid sterility in rice supports a potentially important role of ancestral polymorphisms under balancing selection for the evolution of plant gene-flow barriers.

## Methods

### Biological materials and growth conditions

The geographical origins of the *C. grandiflora*, *C. rubella* and *C. orientalis* accessions used in this study and generation of the RIL population have been described previously^26^,^27^,^47^. Additional *C. grandiflora* populations were collected at the locations indicated in Supplementary Table 4.

The NIL segregating for the two incompatible loci was generated by introgressing the corresponding *Cg926* alleles into a *Cr1504* background by four rounds of backcrossing. Its genotype is given in Supplementary Table 2. All the plants were grown under long day conditions (16 h light, 8 h dark) at 70% humidity with a light level of 150 μmol m^−2^ s^−1^. As default, the temperature cycle was 22 °C during the day and 18 °C during the night. The temperature rescue experiments were conducted at the indicated temperature without any diurnal fluctuations.

### Molecular cloning and plant transformation

The *35S::CrNPR1* and *35S::CgNPR1* constructs were generated by amplifying *NPR1* open reading frames (ORFs) from NIL(*2gg; 7rr*) and NIL(*2rr; 7rr*) seedling cDNA, respectively, using the primers oAS1036 and oAS1037. After subcloning PCR products into the pGEM-T vector (Promega), the *NPR1* ORFs were sequenced and transferred into the plant transformation vector ML595 (a modified version of pGPTVBAR, ref. 48) at a PacI site located between a cauliflower mosaic virus *35S* promoter and terminator.

To downregulate the expression of the *CrRPP5* gene in the incompatible hybrids, a microRNA-induced gene silencing construct was generated as described^36^. A 329-bp fragment of *CrRPP5* cDNA was fused to the recognition site of the miR173 through PCR amplification using the primers oAS1137 and oAS1138. The resulting PCR product, *173ts:RPP5,* was digested and transferred into the PacI site of ML595 as described above.

The *35S::173tsRPP5*, *35S::CrNPR1* and *35S::CgNPR1* constructs were transformed into NIL(*2gg; 7rr*) plants by floral dip^49^.

### Phenotypic characterization

Palisade sub-epidermal cell sizes were measured as previously described^50^. Briefly, entire leaves were fixed overnight at 4 °C in formalin/acetic acid/alcohol and dehydrated through a series of 70, 80, 90 and 100% ethanol, with 5-min incubation per step. The samples were then transferred into acetone and incubated for 5 min at 95°C. Finally the samples were cleared overnight in a chloral hydrate solution (200 g chloral hydrate, 20 g glycerol and 50 ml water). For observation, the samples were mounted in the chloral hydrate solution and imaged under differential interference contrast on an Olympus BX51 microscope using an AxioCam ICc3 camera (Zeiss). These images were used to determine cell area in Image J.

The presence of cell death was studied by staining whole leaves with lactophenol Trypan blue as previously described^51^. Briefly, leaves were boiled in staining solution (6 vols ethanol, 1 vol water, 1 vol lactic acid, 1 vol glycerol, 1 vol phenol, Trypan blue 0.067%) for 2 min and destained in chloral hydrate (2.5 g ml^−1^). Cleared leaves were mounted in chloral hydrate and imaged using an Olympus BX51 microscope and an AxioCam ICc3 camera (Zeiss).

### QTL and fine mapping

QTL mapping was based on the previously described genotypes of the *Cr1504*x*Cg926* RILs^27^. QTL analysis and testing for genetic interaction were performed using the R/QTL package adds-on implemented in the open-source statistical software R (http://www.R-project.org/)^52^,^53^. Ten plants for each of the 142 RILS were scored for the severity of the hybrid incompatibility phenotype from 0 (normal growth) to 3 (strongly stunted growth). LOD scores were calculated with the Multiple QTL Mapping (MQM) function of the R/QTL package using the phenotypic mean value for each RIL and assuming a normal distribution of the phenotypic values. An unsupervised cofactor selection through backward elimination using a cofactor significance of 0.015 was conducted to identify informative markers that needed to be accounted for in the model of the MQM scan. Genome-wide permutations (1,000 permutations) were used to assess the LOD significance threshold (*α*=0.05). The two-LOD score interval was used to determine the position of each QTL within 95% confidence. Additive effect and the percentage of variance explained by each QTL were determined using the fitqtl function with the formula: *y*∼Q1+Q2+Q1:Q2. The effect of the interaction between the two QTL was visualized using the effectplot R function. Genetic interactions between the QTL identified were tested using the scantwo function with an Haley–Knott regression.

The PCR-based markers used for fine mapping were retrieved from the whole genome resequencing of *Cr1504* and *Cg926* (Supplementary Table 5). The genetic mapping of the genes underlying QTL2 and QTL7 was performed using NIL(*2rg; 7rr*) and NIL(*2gg; 7rg*) plants, respectively. The progenies of these plants were screened for recombination breakpoints between the markers HiB1 and HiB8 and between HiG1 and HiG6, respectively. In total 667 and 1,117 plants were screened to narrow down the region underlying the QTL2 and QTL7. For critical recombinants, the genotype at the incompatible locus was verified by studying the segregation of the stunted growth within their progenies.

### Quantitative reverse transcription–PCR

Total mRNA was extracted from young leaves of 20-day-old seedling, using trizol (Life Technologies), treated with Turbo DNAase (Ambion) and reverse transcribed with the Superscript III Reverse Transcriptase (Invitrogen). This template was then used to quantify relative abundance of specific transcripts using the primers described in Supplementary Table 5, the SensiMix SYBR Low-ROX kit (Bioline) and a LightCycler 480 (Roche). Each data point was based on three technical replicates each from three biological replicates.

### Allele frequencies, sequence and population-genetic analyses

To reconstitute the phylogeny at the *NPR1* and the *RPP5* loci, fragments of about 700 bp and 800 bp, respectively, were resequenced in several *C. grandiflora* and *C .rubella* accessions using the primer pairs oAS1129-oAS1130 and oAS873-oAS1123. The *C. orientalis* sequences were retrieved from the previously described whole genome resequencing of 10 individuals^47^. The presence or absence of the *RPP5* gene was tested by PCR using the primers oAS873 and oAS1123. The phylogenetic relationship between the different sequences was investigated by constructing neighbour-joining trees with MEGA5 (ref. 54) using maximum composite likelihood method. Haplotype networks were reconstituted using Haplotype Viewer (http://www.cibiv.at/~greg/haploviewer).

Allele frequencies at the *NPR1* and the *RPP5* loci were estimated by PCR genotyping *C. grandiflora* populations with the primer pair oAS1129-oAS1130 for *NPR1*, and oAS873-oAS1123 and oAS839-1133 for *RPP5*. The primer pair oAS1129-1130 reveals a 39-nt deletion within the exon3 of *NPR1*. Primer pair oAS873-oAS1123 amplifies 1.1 kb at the 3′ end of the *RPP5* ORF, while oAS839-113 reveals the 7.6 kb deletion of the RPP5 gene. The *C. grandiflora* seeds were collected around the Zagori Mountains in Greece leaving at least 1 m between the collected plants (see ‘Biological materials and growth conditions’ section above). Four progenies of each of these plants were independently genotyped to estimated allele frequencies. The C. *grandiflora* populations sampled were plotted on an OpenStreetMap (https://www.openstreetmap.org/copyright) based map using the R package ggmap. OpenStreetMap is available under the Open Database License (ODbL) v1.0 (http://opendatacommons.org/licenses/odbl/1.0/) as OA-SA. The total number of plants genotyped is indicated in Fig. 3. Note that for the *RPP5* locus, we have not been able to amplify a fragment in all of the *C. grandiflora* plants, which is likely due to the high sequence and structural variability at the *RPP5* locus. We have called the missing fraction of haplotypes ‘other *RPP*’. Of note, this effect may have lead to an overestimation of the *CrRPP5* and *CgRPP5* alleles, if other *RPP5* alleles went undetected in heterozygous plants.

The data for population-genetic analysis and association testing are a subset of a whole genome population resequencing study of within-population variation (Lee *et al.*, manuscript in preparation). Briefly, 178 genotypes were derived from *C. grandiflora* population 9 from Epiros, Zagory mountains, Greece^55^ and DNA was extracted by either a CTAB-based protocol or by DNeasy Plant Mini Kit (Qiagen). We obtained whole genome sequences from each individual through 100 cycles of paired end sequencing in a Hi-seq 2000 with Truseq libraries (Illumina). Three individuals were sequenced per lane. Reads were mapped to the *C. rubella* reference genome^23^ with Stampy v.1.0.19. After bioinformatic processing with Picard tools, we realigned reads around putative indels with GATK RealignerTargetCreator and IndelRealigner and then compressed the resulting bams with GATK ReduceReads. Raw single nucleotide polymorphism (SNP) calls were generated by joint calling of all 178 samples in GATK v2.81 UnifiedGenotyper. We subsequently followed GATK Best Practices for Variant Quality Recalibration using a high confidence subset of the raw calls generated by filtering SNPs for concordance with common variants (>0.11) in a species-wide sample of *C. grandiflora*^56^, as well as suspect realignments (transposable elements, centromeres, 600-bp intervals containing extreme Hardy–Weinberg deviations, 1-kb intervals that showed evidence of three or more SNPs in a reference-to-reference mapping of 150-bp-paired end reads from the reference genome line).

Using our filtered polymorphism data, we generated diversity summary statistics using custom python scripts across chromosome 2 in the sample of 178 *C. grandiflora* individuals (downsampled to 320 alleles, which allowed retention of 94.2% of sites) in 500-bp windows with 250-bp overlaps^57^ or 50-SNP windows^58^. Linkage disequilibrium was calculated using Haploview^59^ for unphased SNPs with minor allele frequency >5% in a 30-kb region centred around *NPR1*, for all pairs of SNPs.

### Crossing experiments

*C. grandiflora* × *C. rubella* crosses were generated by randomly crossing different *C. grandiflora* individuals originating from various Greek populations with several *C. rubella* accessions (see crossing scheme in Supplementary Fig. 7). The F1 plants were genotyped at the *NPR1* and *RPP5* loci. The *NPR1* locus was genotyped using the primers oAS1129 and oAS1130 (Supplementary Table 5). The deletion of *RPP5* locus was genotyped using the primer pairs oAS839-oAS1133 and oAS839-oAS1276, while its presence was determined using the primer pairs oAS873 and oAS1123 (Supplementary Table 5). The F2 progenies of these crosses were phenotyped for the segregation of the stunted growth phenotype and genotyped at *NPR1* (primers oAS1129 and oAS1130), as well as at *RPP5* using the very tightly linked molecular marker HiG2 to enable genotyping also for populations in which no *RPP5*-presence allele segregated (Supplementary Table 5). The marker HiG2 was chosen as it was the closest marker from *RPP5* for which the underlying polymorphism was conserved between the different *C. grandiflora* and *C. rubella* parents used for the crosses. *C. rubella 1504* × *C. orientalis 1983* and *C. orientalis 1983* × *C. grandiflora 926* hybrids were obtained by ovule rescue as previously described^60^. Their F2 progenies were phenotyped and genotyped as indicated above.

### Transcriptome analysis

Transcriptome sequencing was done for 99 samples from one *C. grandiflora* population, grown in a growth chamber at 22 °C with 16-h photoperiod. We extracted RNA using the Spectrum Plant Total RNA Kit (Sigma) from leaf tissue collected and flash frozen 5 weeks after germination. RNA was sequenced in two Illumina Hi-Seq flow cells with eight samples per lane. Reads were 100-bp long and paired end. We mapped RNA with Stampy 1.0.21 (ref. 61) to a custom reference genome constructed of exons and untranslated regions for each gene, using annotations from ref. 23. Expression levels were measured as the number of paired fragments mapping to each gene using the HTSeq.scripts.count feature of HTSeq^62^, and read counts were normalized for sequencing depth by dividing each count by the median count for that individual. Genes with a median un-normalized expression level of <5 reads per individual or with very low variation in expression between individuals (coefficient of variation<0.1) were removed from further analysis leaving 18,692 genes in the analysis.

Genes were linked to the *NPR1* 39-nt InDel genotype by testing for an association between *NPR1* InDel genotype and gene-expression levels using a Kruskal–Wallis test, because expression data were not normally distributed. While none of the associations were significant after controlling for false discovery rates using *Q* value^63^, we included the 1,000 genes with the lowest *P* values for association in subsequent analyses.

### MapMan and MASTA analysis

Identification of overrepresented gene categories within the 1,000 genes with the lowest *P* values for association with the *NPR1* genotype was done using the MapMan ontology^39^ and Fisher's exact test in R. The *C. rubella* MapMan mappings are available at http://mapman.gabipd.org. The same 1,000 genes were compared with the top 200 up- and downregulated genes of each experiment present in the MASTA data set of *A. thaliana* microarrays^40^. Only *C. rubella* genes with correspondence to *A. thaliana* microarray based on annotations in Phytozome version 8 (ref. 64) were considered, all other genes were filtered out from the MASTA data set. Top overlapping categories based on MASTA classification were determined using a Wilcoxon rank-sum test with overlap counts; *P* values were Bonferroni corrected for multiple testing.

### Statistical analysis

Within each experiment, plants of the different genotypes were randomly assigned to positions in the trays, and trays were rotated once per week in the growth rooms. No blinding was performed. The statistical analysis was performed using R or Microsoft Excel 7. Data were presented as mean±s.e.m. To analyse the difference between genotypes Tukey’s honest significant difference test was carried out using agricolae package add-ons implemented in R software. To compare genotype frequencies between different phenotype classes, the *χ*^2^-test was used. *P* values<0.05 were considered statistically significant.

## Additional information

**Accession codes:** Sequences of *NPR1* and *RPP5* alleles from the *C. grandiflora* × *C. rubella* RIL population have been deposited in NCBI GenBank nucleotide database under accession codes KT163438 to KT163440. Transcriptome and genome-resequencing data of *C. grandiflora* individuals have been deposited in NCBI under accession code PRJNA275635.

**How to cite this article:** Sicard, A. *et al.* Divergent sorting of a balanced ancestral polymorphism underlies the establishment of gene-flow barriers in *Capsella*. *Nat. Commun.* 6:7960 doi: 10.1038/ncomms8960 (2015).

## Supplementary Material

Supplementary Information Supplementary Figures 1-9, Supplementary Tables 1-5, Supplementary Discussion and Supplementary Reference
