# Supplementary material for: Divergent sorting of a balanced ancestral polymorphism underlies the establishment of gene-flow barriers in Capsella
Source: Nat Commun. 2015 Aug 13;6:7960. doi: 10.1038/ncomms8960 (PMC4539569; doi:10.1038/ncomms8960)
Supplement: Supplementary Information — Supplementary Figures 1-9, Supplementary Tables 1-5, Supplementary Discussion and Supplementary Reference [file ncomms8960-s1.pdf]

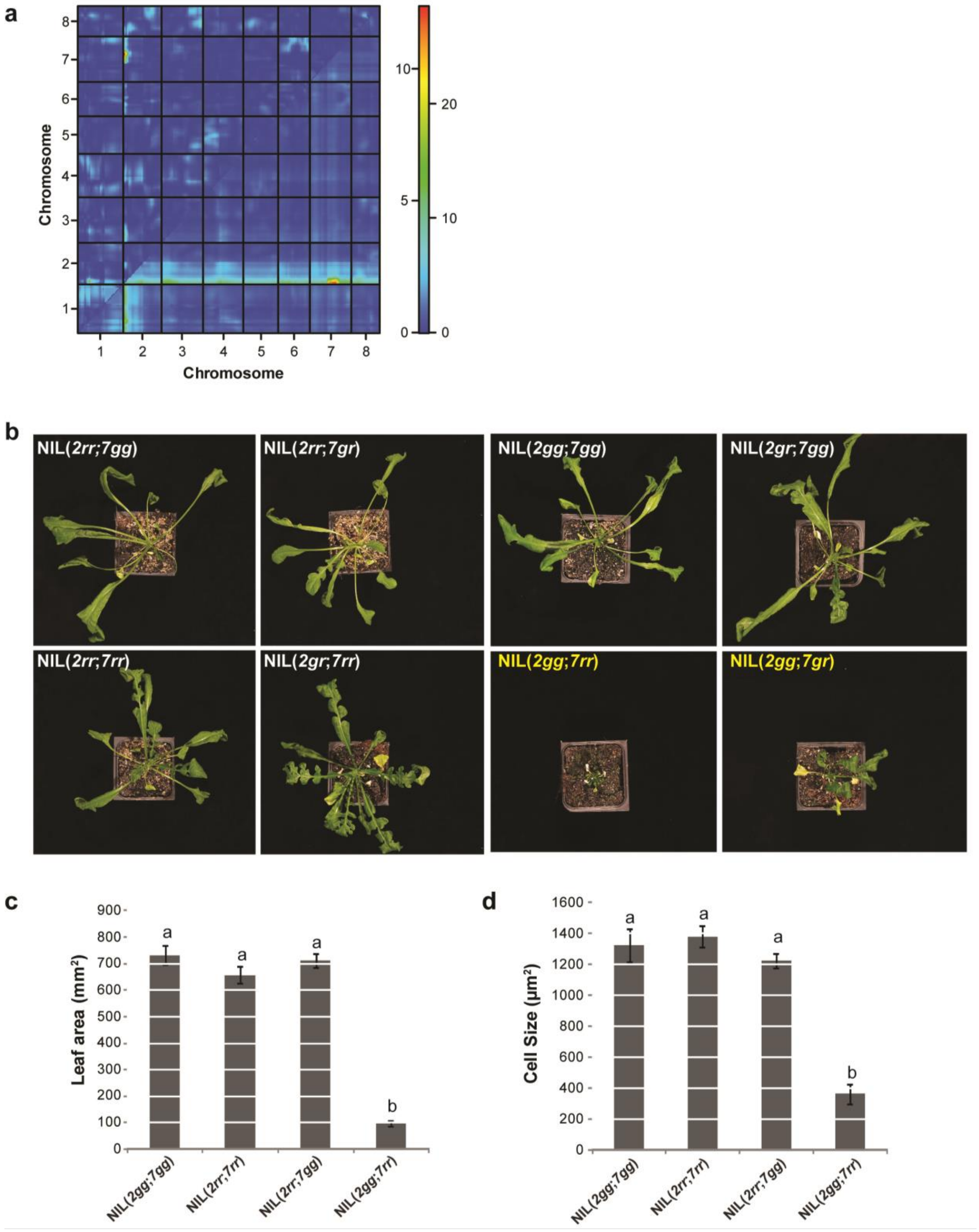

Supplementary Figure 1: Characterisation of the incompatible interaction in *C. grandiflora* x *C. rubella* hybrids.

a Results of two-dimensional QTL mapping for the incompatible phenotype. The full LOD score is shown below the diagonal, the interaction LOD score above. Colour scale refers to interaction LOD

score (left) and full LOD score (right). This QTL experiment has been performed using the phenotypic average values of 10 replicates for 142 RILs.

b Phenotypes of NIL plants with the indicated genotypes. Yellow font indicates incompatible phenotypes.

c,d Leaf area (**c**) and leaf-cell area (**d**) in the indicated genotypes. Values are mean ± s.e.m. of 10 and 4 leaves respectively per genotype, respectively. Letters indicate significant differences as determined by Tukey's HSD test (α =0.05).

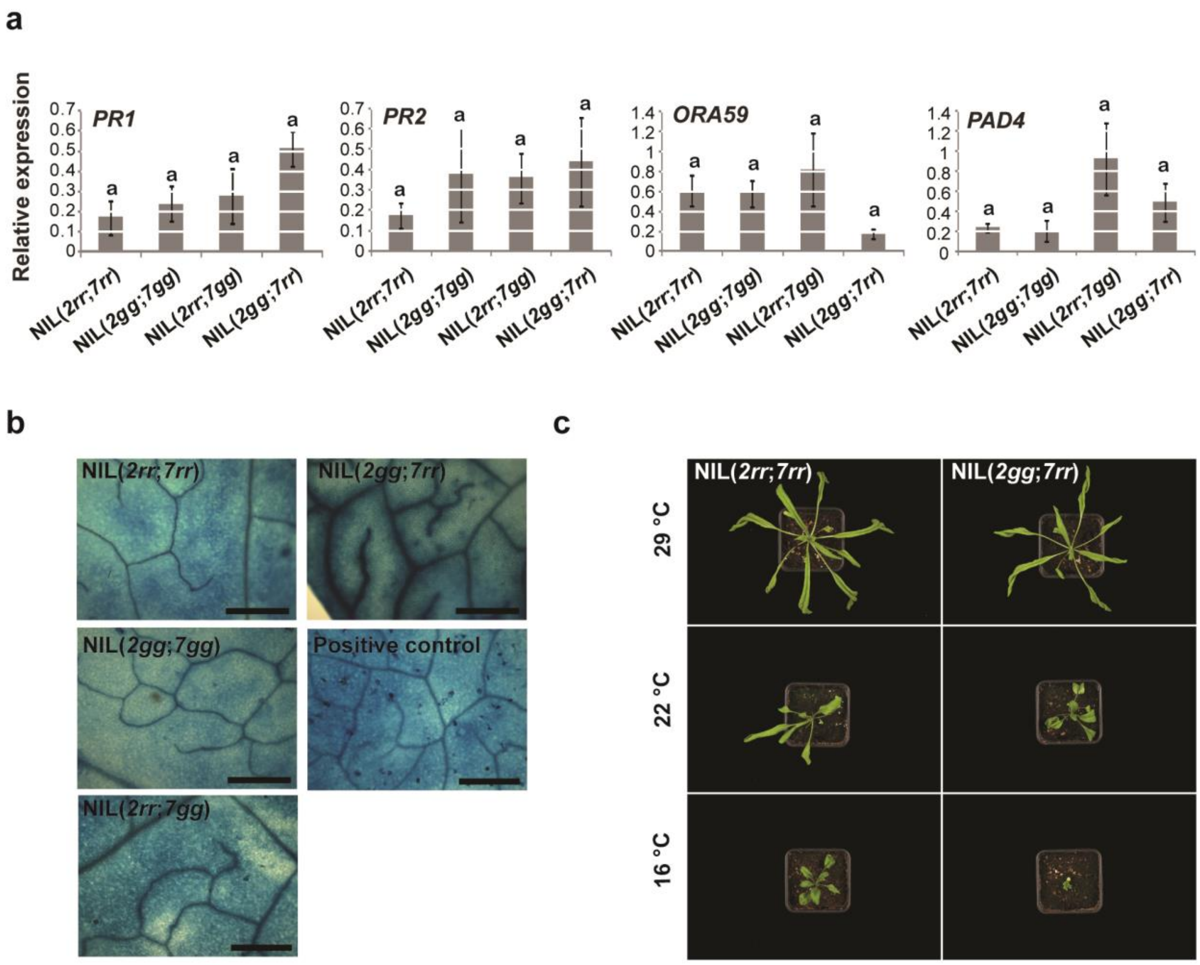

Supplementary Figure 2: Characterization of the constitutive immune response in incompatible hybrids.

a Expression of the indicated immune-response markers determined by qRT-PCR normalized to *Capsella TUB6*. Mean ± s.e.m. of three biological replicates is shown. Letters indicate significant differences as determined by Tukey's HSD test (α =0.05).

b Trypan Blue staining of leaves from the indicated genotype fails to detect ectopic necrosis in incompatible hybrids. Positive control is an early senescent leaf.

c The incompatible phenotype in NIL(*2gg; 7rr*) plants (right) can be rescued by growth at elevated temperatures as compared to compatible control plants (left).

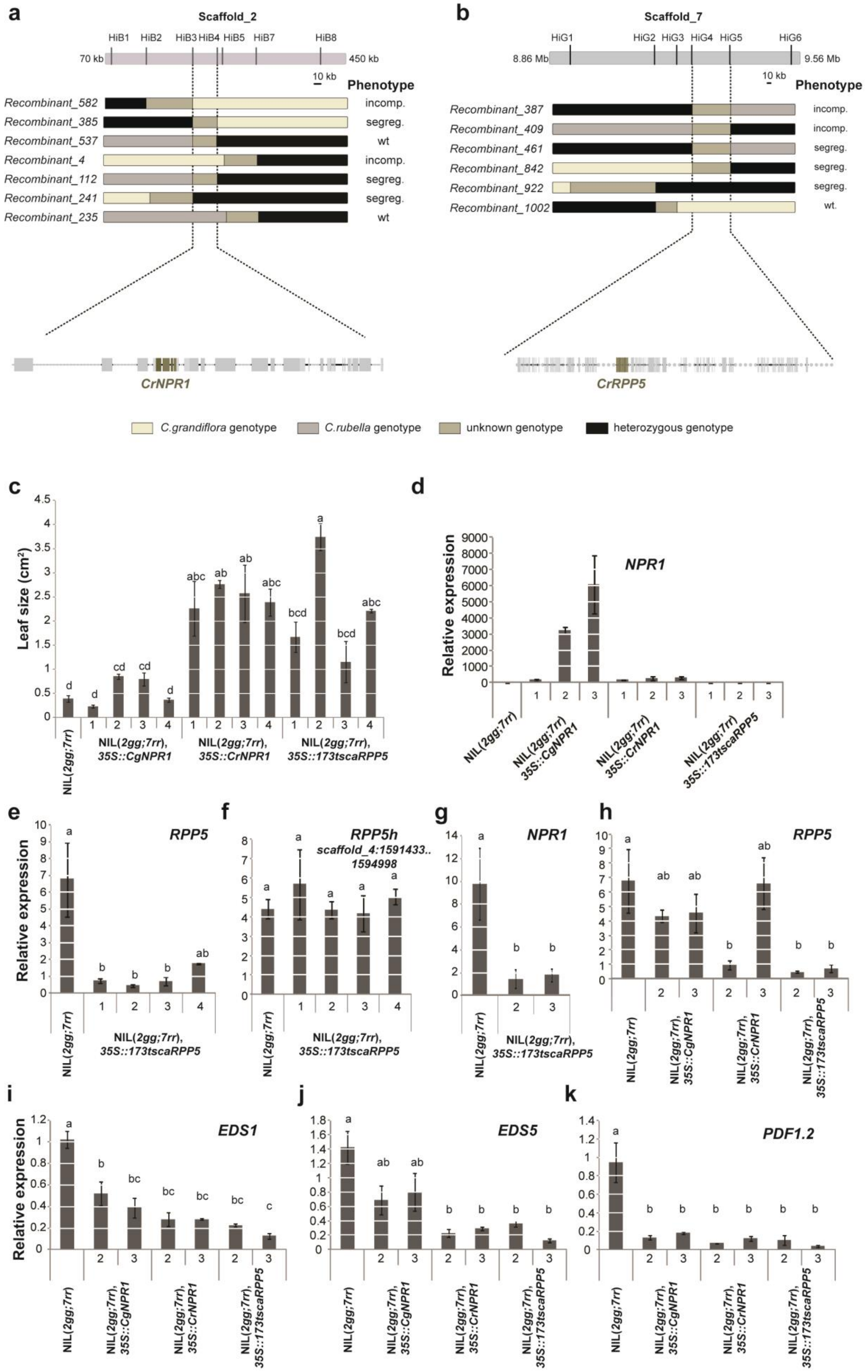
a
Scaffold_2
HiB1 HiB2 HiB3 HiB4 HiB5 HiB7 HiB8
70 kb
450 kb
10 kb
Phenotype
Recombinant_582 incomp.
Recombinant_385 segreg.
Recombinant_537 wt
Recombinant_4 incomp.
Recombinant_112 segreg.
Recombinant_241 segreg.
Recombinant_235 wt
CrNPR1
b
Scaffold_7
HiG1 HiG2 HiG3 HiG4 HiG5 HiG6
8.86 Mb
9.56 Mb
10 kb
Phenotype
Recombinant_387 incomp.
Recombinant_409 incomp.
Recombinant_461 segreg.
Recombinant_842 segreg.
Recombinant_922 segreg.
Recombinant_1002 wt.
CrRPP5
C.grandiflora genotype
C.rubella genotype
unknown genotype
heterozygous genotype
c
Leaf size (cm²)
NIL(2gg;7rr)
NIL(2gg;7rr), 35S::CgNPR1
NIL(2gg;7rr), 35S::CrNPR1
NIL(2gg;7rr), 35S::173tscaRPP5
d
Relative expression
NPR1
NIL(2gg;7rr)
NIL(2gg;7rr) 35S::CgNPR1
NIL(2gg;7rr) 35S::CrNPR1
NIL(2gg;7rr) 35S::173tscaRPP5
e
RPP5
Relative expression
NIL(2gg;7rr)
NIL(2gg;7rr), 35S::173tscaRPP5
f
RPP5h
scaffold_4:1591433..
1594998
NIL(2gg;7rr)
NIL(2gg;7rr), 35S::173tscaRPP5
g
NPR1
NIL(2gg;7rr)
NIL(2gg;7rr), 35S::173tscaRPP5
h
RPP5
NIL(2gg;7rr)
NIL(2gg;7rr), 35S::CgNPR1
NIL(2gg;7rr), 35S::CrNPR1
NIL(2gg;7rr), 35S::173tscaRPP5
i
EDS1
Relative expression
j
EDS5
k
PDF1.2
NIL(2gg;7rr)
NIL(2gg;7rr), 35S::CgNPR1
NIL(2gg;7rr), 35S::CrNPR1
NIL(2gg;7rr), 35S::173tscaRPP5

Supplementary Figure 3: Fine-mapping of the incompatible loci and transgenic rescue of the incompatible phenotype.

a, b Location of markers used for mapping (top), genotypes and associated phenotypes of informative recombinants (middle) and annotated genome structure in the QTL2 (**a**) and QTL7 regions (**b**). Rectangles in the genome annotation represent exons, solid lines are introns and dotted lines show intergenic sequences.

c Leaf size in the indicated genotypes. Values are means ± s.e.m. from 4 leaves per genotypes. Numbers indicated independent transgenic lines for the same construct. Letters indicate significant differences as determined by Tukey's HSD test (α =0.05).

d-k Expression of *NPR1* (**d,g**), *RPP5* (**e,h**) and its closest *Capsella* paralogue *RPP5h* (**f**), and of immune-response markers *EDS1* (**i**), *EDS5* (**j**) and *PDF1.2* (**k**) determined by qRT-PCR normalized to *Capsella TUB6*. Mean ± s.e.m. of three (**d-f**) or four (**g-k**) biological replicates is shown. Letters indicate significant differences as determined by Tukey's HSD test (α =0.05). Numbers indicated independent transgenic lines for the same construct as in (**c**).

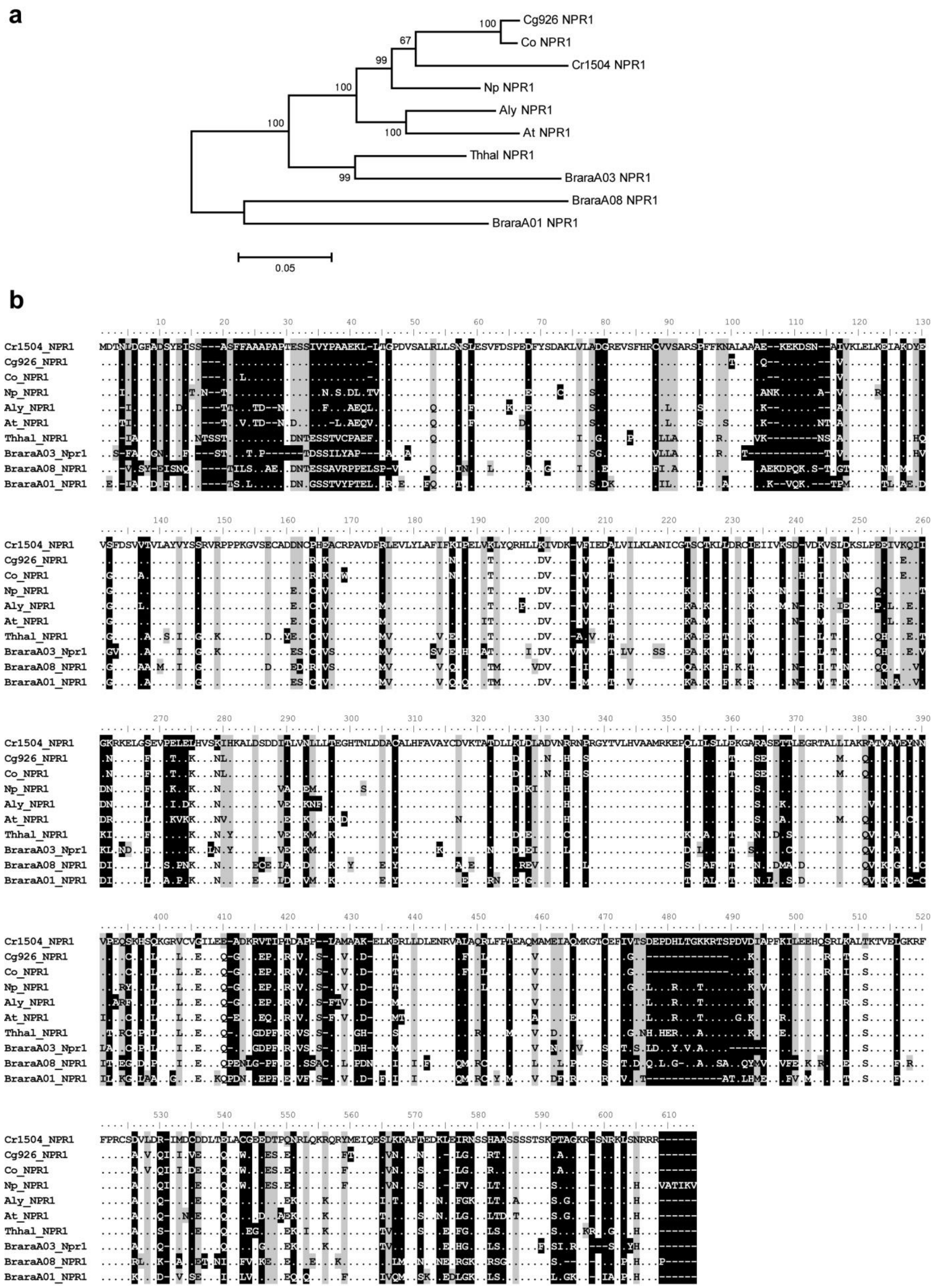

Supplementary Figure 4: ***NPR1*** **phylogeny in Brassicaceae**

a, b Phylogenetic tree (**a**) and protein sequence alignment (**b**) of NPR1 orthologues from different Brassicaceae. Identical amino acids are indicated by dots; conservative changes by grey, non-conservative changes by black shading. Aly: *Arabidopsis lyrata*, Ath: *A. thaliana*, Brara: *Brassica rapa*, Np: *Neslia paniculata*, Thhal: *Thelungiella halophila*.

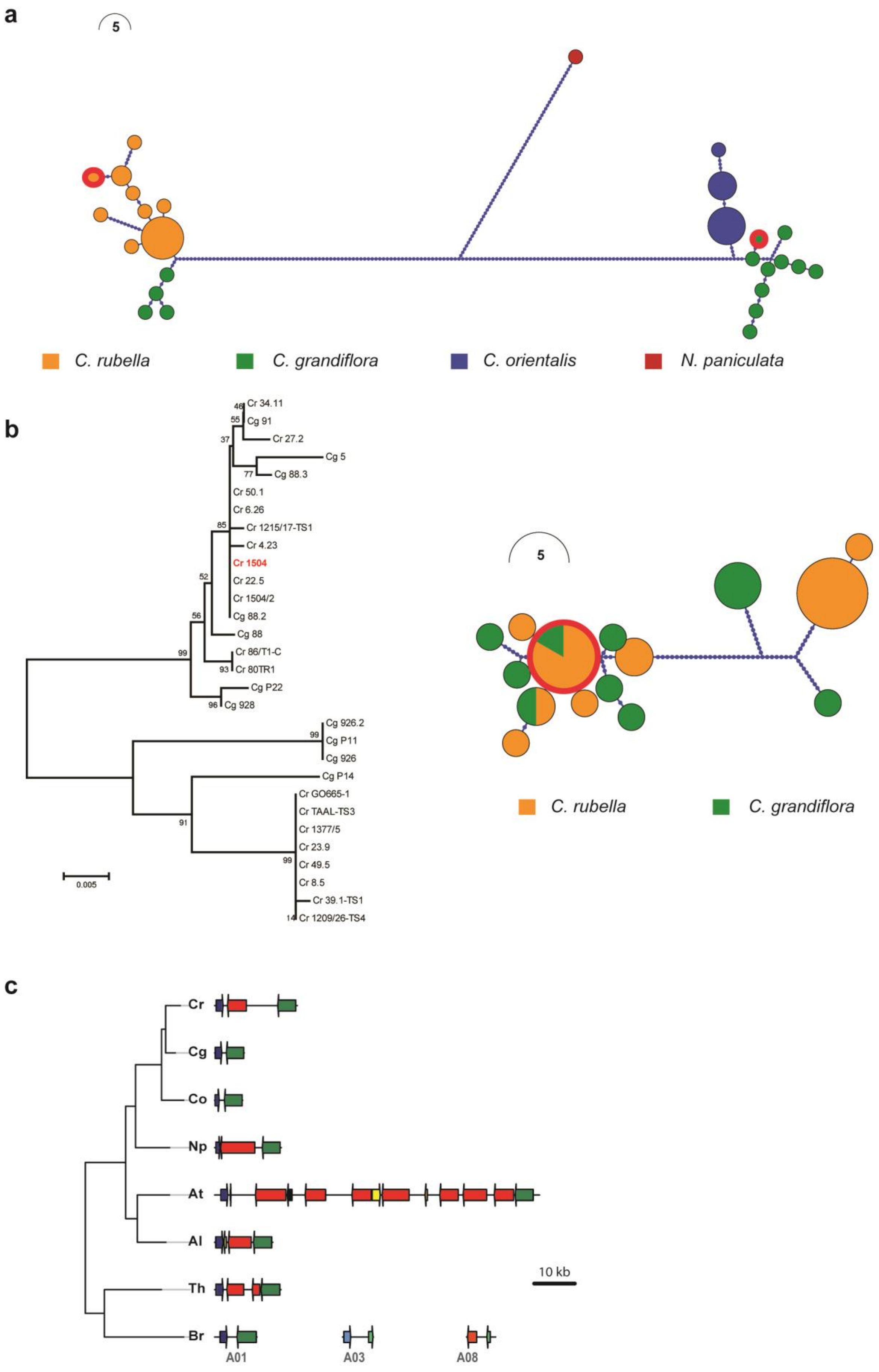
a
5
C. rubella
C. grandiflora
C. orientalis
N. paniculata
b
Cr 34.11
Cg 91
Cr 27.2
Cg 5
Cg 88.3
Cr 50.1
Cr 6.26
Cr 1215/17-TS1
Cr 4.23
Cr 1504
Cr 22.5
Cr 1504/2
Cg 88.2
Cg 88
Cr 86/T1-C
Cr 80TR1
Cg P22
Cg 928
Cg 926.2
Cg P11
Cg 926
Cg P14
Cr GO665-1
Cr TAAL-TS3
Cr 1377/5
Cr 23.9
Cr 49.5
Cr 8.5
Cr 39.1-TS1
Cr 1209/26-TS4
0.005
5
C. rubella
C. grandiflora
c
Cr
Cg
Co
Np
At
Al
Th
Br
10 kb
A01
A03
A08

Supplementary Figure 5: Haplotype structure of *NPR1* and *RPP5* in *C. grandiflora* and *C. rubella*.

a Haplotype network of *NPR1* in *C. grandiflora* (green), *C. rubella* (yellow), *C. orientalis* (blue) and *N. paniculata* (red). Red outlines indicate the *NPR1* haplotypes segregating in the RIL population. Analysis is based on resequencing of a 700 bp fragment.

b Neighbor-joinng phylogenetic tree of *RPP5* haplotypes from different *C. grandiflora* and *C. rubella* accessions (left) and corresponding haplotype network (right). Analysis is based on resequencing of an 800 bp fragment. Numbers indicated bootstrap support from 1,000 runs.

c Genome structure of *RPP5* region in Brassicaceae. Colours indicate syntenic genes. The phylogenetic tree shows known evolutionary relationships between the species. Corresponding regions from the three *B. rapa* subgenomes are shown.

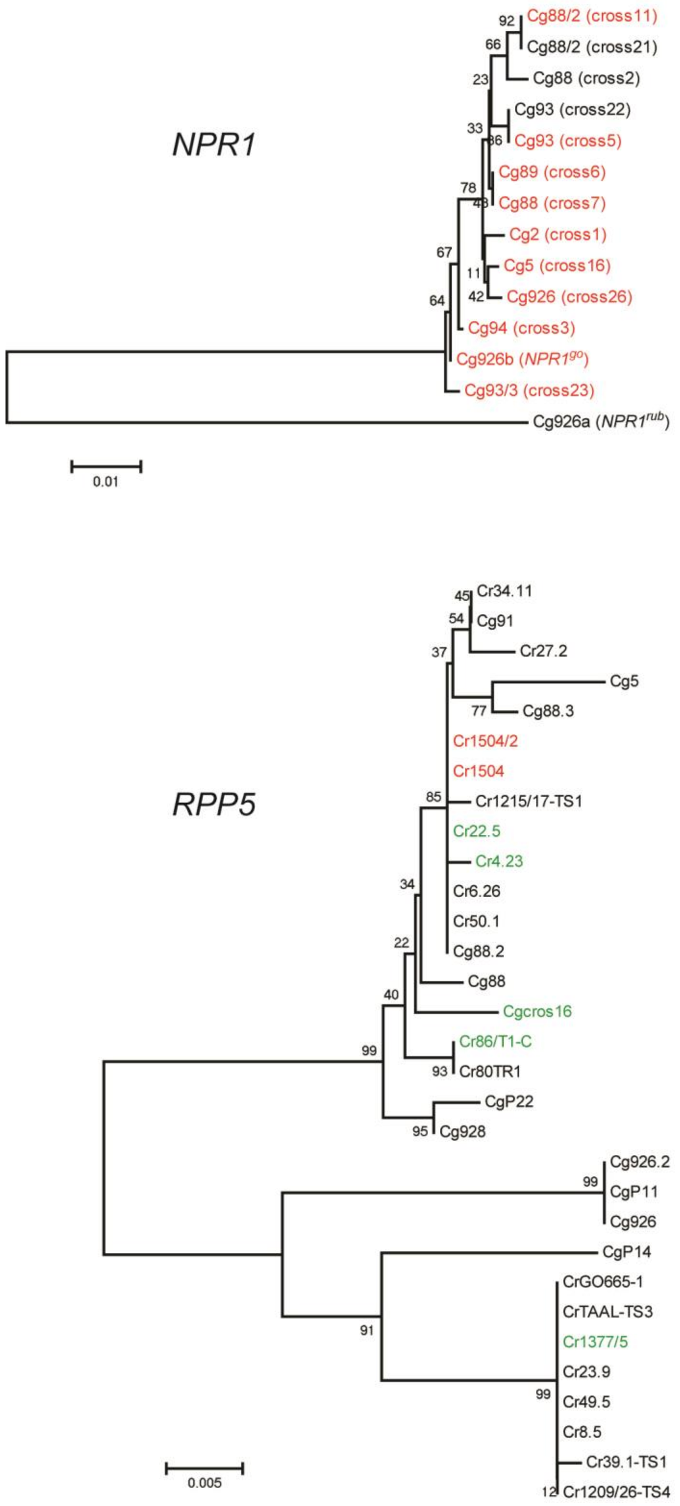

Supplementary Figure 6: Extended *NPR1* and *RPP5* phylogenies.

Phylogenetic trees for *NPR1* (top) and *RPP5* (bottom) including additional accessions used for crosses in Supplementary Figure 7. Incompatible haplotypes are indicated in red, compatible ones in blue and haplotypes not tested in grey. Analyses are based on resequencing of 700 bp (*NPR1*) and 800 bp fragments (*RPP5*). Numbers indicated bootstrap support from 1,000 runs. Red font indicates the alleles that have been tested in crossing experiments and shown to be incompatibles; green font

indicates alleles that have been tested in crossing experiments and shown to be compatible; and black font indicates the alleles that have not been tested.

a

Cross number	*C. grandiflora* accession	*C. rubella* accession	F1 genotype*	% incompatible phenotype	total	*n* strongly incompatible	moderately incompatible
1	*Cg 2*	*Cr 1504/2*	*2gr;7gr*	9.24	119	1	10
5	*Cg 93*	*Cr 1504/2*	*2gr;7gr*	22.12	113	7	18
6	*Cg 89*	*Cr 1504/2*	*2gr;7gr*	32.32	99	9	23
7	*Cg 88*	*Cr 1504/2*	*2gr;7gr*	10.26	117	0	12
11	*Cg 88/2*	*Cr 1504/2*	*2gr;7gr*	20.00	95	0	19
16	*Cg 5*	*Cr 1504*	*2gr;7rr*	8.30	253	0	21
23	*Cg 93/2*	*Cr 1504*	*2gr;7gr*	12.35	251	14	17
26	*Cg 926*	*Cr 1504*	*2gr;7gr*	25.00	148	8	29
30	*Cg 926/2*	*Cr 1504*	*2gr;7gr*	18.60	129	4	20
2	*Cg 88*	*Cr 4.23*	*2gr;7gr*	0	110	0	0
9	*Cg 88/3*	*Cr 4.23*	*2gr;7gr*	20.25	79	8	8
4	*Cg 926/3*	*Cr 86IT1-C*	*2rr;7rg*	0	97	0	0
12	*Cg 926/4*	*Cr 22.5*	*2rr;7rg*	0	119	0	0
21	*Cg 88/2*	*Cr 22.5*	*2gr;7gr*	3.88	103	3	1
3	*Cg 94*	*Cr 1GR1-TS1*	*2gr;7gg*	22.03	177	20	19
20	*Cg 83*	*Cr 1GR1-TS1*	*2gr;7gg*	0	132	0	0
24	*Cg 88*	*Cr 1GR1-TS1*	*2gr;7gg*	0	129	0	0
8	*Cg 89*	*Cr 1377/5*	*2gr;7gr*	0	126	0	0
10	*Cg 5*	*Cr 1377/5*	*2gr;7gr*	17.75	169	14	16
13	*Cg 2h*	*Cr 1377/5*	*2gr;7gr*	0	129	0	0
25	*Cg 94*	*Cr 1377/5*	*2gr;7gr*	0	135	0	0
27	*Cg 5/2*	*Cr 1377/5*	*2gr;7gr*	0	138	0	0

* for QTL2, "r" denotes an *NPR1rub* allele, "g" denotes an *NPR1go* allele; for QTL7, "r" denotes the presence of *RPP5*, "g" denotes its absence

b

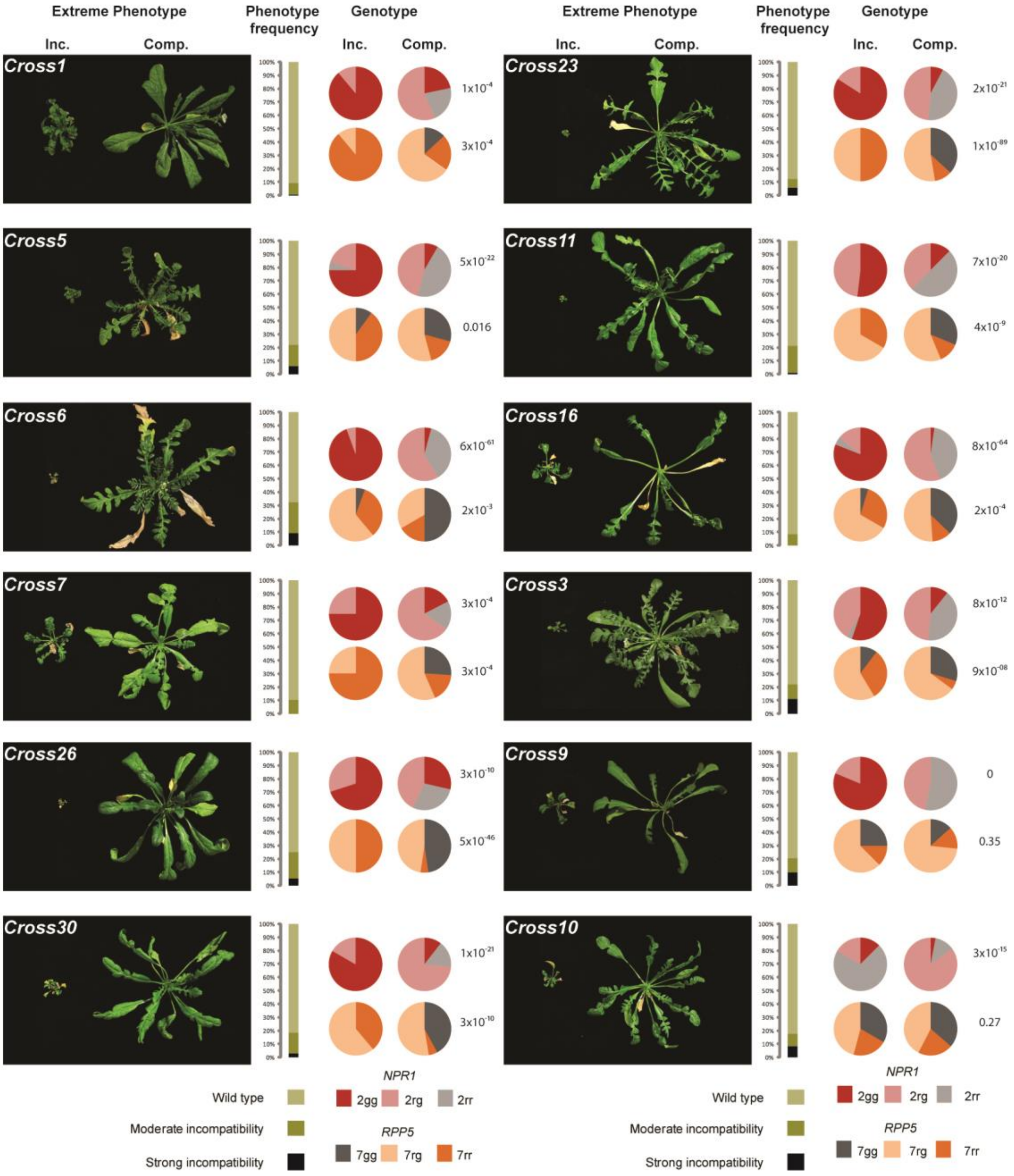

Supplementary Figure 7: Incompatible phenotypes in additional *C. rubella* x *C. grandiflora* crosses.

a Table of additional crosses performed and proportion of incompatible phenotypes in resulting F2 populations. Blue font indicates crosses in which no *RPP5* was segregating. Note that the absence of incompatible phenotypes in crosses 4 and 12 is not informative, as the *C. grandiflora* parental plants of these crosses did not pass on an $NPR1^{go}$ allele to the respective F1 plants. This is due to the *C. grandiflora* accession *Cg926* being heterozygous for $NPR1^{go}/NPR1^{rub}$ (see Fig. 3a).

b Phenotypes and genotypes of compatible and incompatible plants in F2 populations from crosses in (**a**). Numbers indicate the p-values determined by Chi-square test comparing genotype frequencies in the compatible and incompatible cohort. The sample size for the calculation of the phenotype frequency is indicated in figure 7a, n= 16 - 38 and n=8- 44 for the genotype frequency of the compatible and incompatible phenotypic classes respectively.

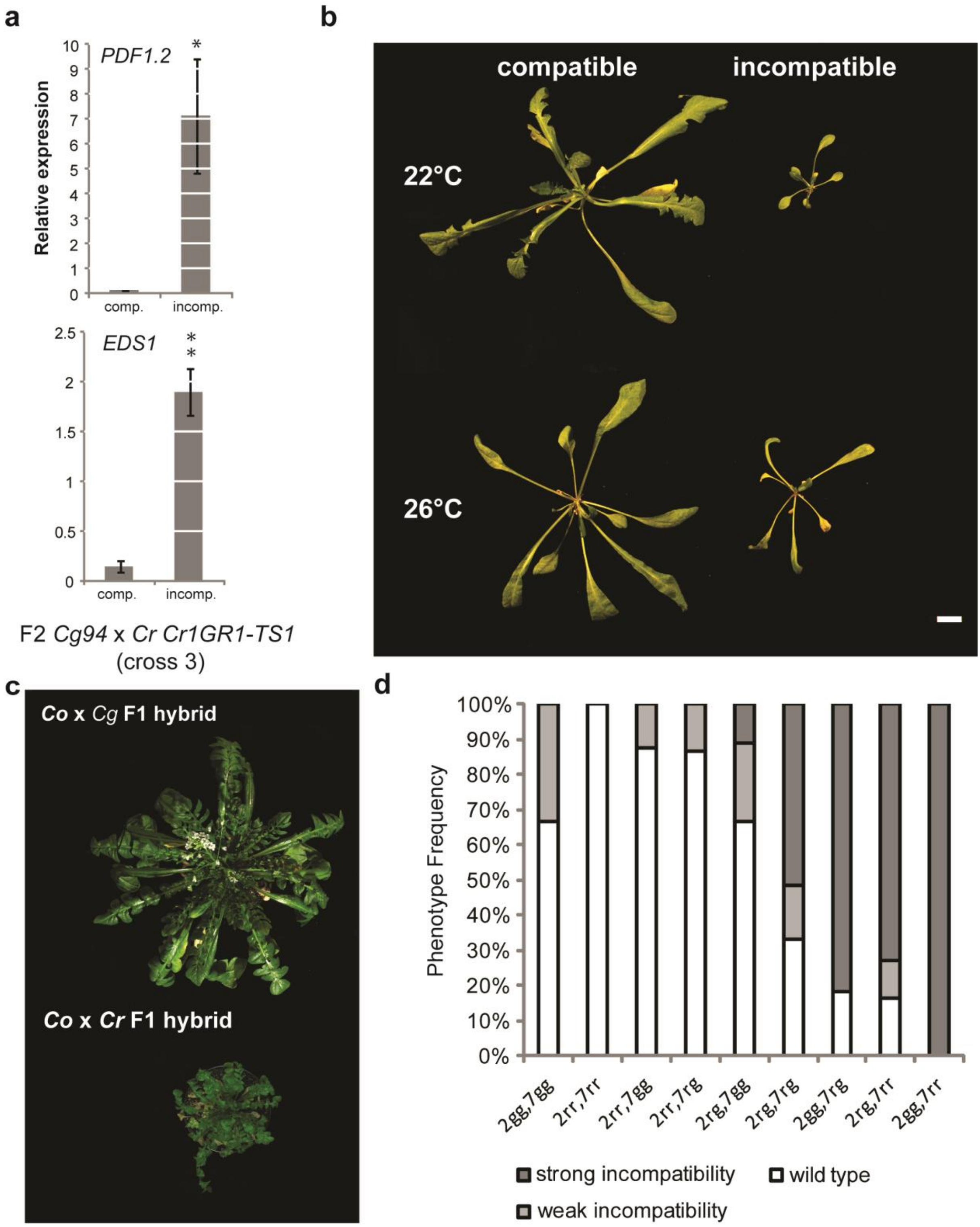

Supplementary Figure 8: Characterization of an additional *NPR1*-linked, but *RPP5*-independent incompatibility between *C. grandiflora* and *C. rubella*, and of the incompatibility between *C. rubella* and *C. orientalis*.

a Expression of the indicated immune-response markers determined by qRT-PCR normalized to *Capsella TUB6* in compatible and incompatible plants from cross 3 in Supplementary Figure 7. Mean

± s.e.m. of three biological replicates is shown. Asterisks indicate significant differences at * $p<0.05$ and ** $p<0.01$ based on a two tailed Student's t-test.

b Growth retardation in incompatible genotypes from cross 3 in Supplementary Figure 7 is alleviated by higher ambient temperatures.

c *C. orientalis Co1983* x *C. rubella Cr1504* (*Co* x *Cr*) F1 hybrids show an incompatible phenotype, whereas *C. orientalis Co1983* x *C. grandiflora Cg926* (*Co* x *Cg*) F1 hybrids do not.

d Phenotype distribution in different genotype classes of F2 plants from the *C. rubella 1504* x *C. orientalis* cross (n=144). For the *NPR1* genotype on chromosome 2, ‘r’ refers to the $NPR1^{rub}$ allele and ‘g’ refers to the $NPR1^{go}$ allele; for the *RPP5* genotype on chromosome 7, ‘r’ refers to the presence of *RPP5*, while ‘g’ refers to the deletion allele.

a

Bin	Name	Elements	Odds ratio	P-value
30.2.11	signalling.receptor kinases.leucine rich repeat XI	209	3.623193	0.0001604
29.4.1.57	protein.postranslational modification.kinase.receptor like cytoplasmatic kinase VII	318	2.6974307	0.0018602
29.4.1	protein.postranslational modification.kinase	326	2.62425436	0.0019645
20.1	stress.biotic	615	2.05602373	0.0038048
20	stress	1100	1.68163609	0.0154877
26.8	misc.nitrilases, *nitrile lyases, berberine bridge enzymes, reticuline oxidases, troponine reductases	102	3.69128104	0.0167027
29.4	protein.postranslational modification	1027	1.61747364	0.0488519

b

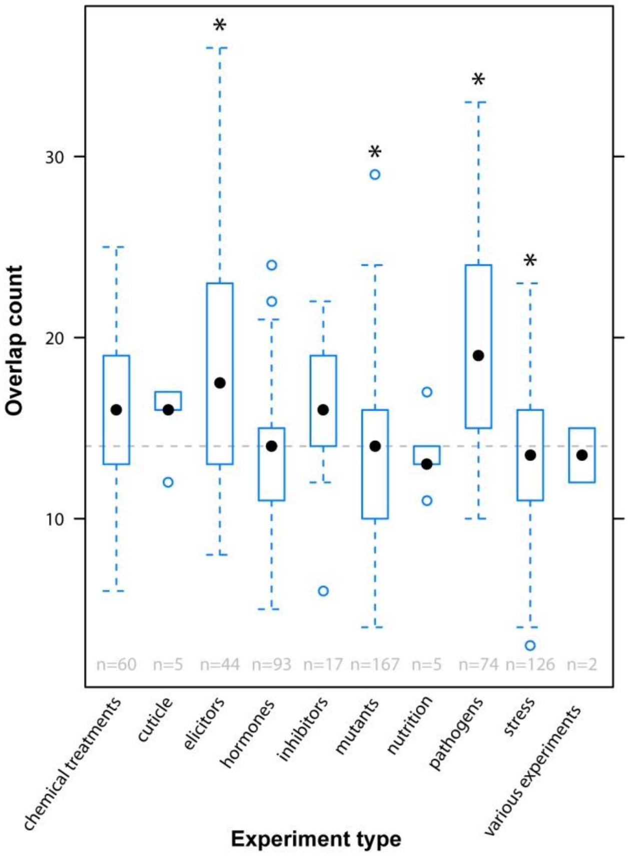

c

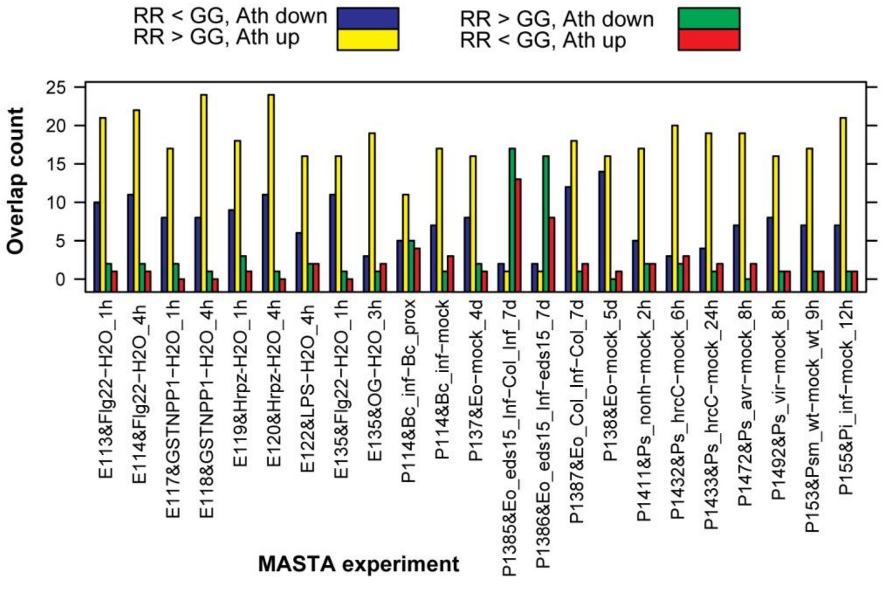

Supplementary Figure 9: Transcriptome effects of different *NPR1* allele clades.

a Table of overrepresented ($p<0.05$) MapMan categories amongst the 1000 genes with the lowest p-values when testing for association between gene expression and *NPR1* genotype. Categories were identified using a Fisher test. Bin and Name describe the categories, elements gives the number of genes of the category. P-values are BH corrected.

b Distributions of gene-overlap counts between the 1000 genes with the lowest p-values when testing for association between gene expression and *NPR1* genotype and *Arabiodpsis thaliana* microarray experiments grouped according to experiment type (n= 2-167). Overlaps were determined using MASTA; experiment type is based on MASTA classification. Asterisks mark significant difference from overlap counts with all experiments at $p<0.05$ based on Wilcoxon rank-sum test. n indicate the number of experiments per category. Pathogen experiments were the most strongly significant ones ($p<1e-11$) Dashed grey line represents median overlap for all experiments.

Supplementary Table 1: Summary results of the QTL mapping for stunted growth in the *Cg926* x *Cr1504* RILs.

QTL	location in cM (closest marker)	LOD	2LOD Confidece interval in cM (surronding markers)	Additive effect	Phenotypic variance explained (%)
QTL2	0	20.522	0-5 (cap5-1g63)	-0.36	41.261
QTL7	35	11.694	30-45 (g06-g08)	0.26	15.462

The additive effect was calculated as half of the difference of the phenotypic averages between Cr and Cg homozygote genotypes derived from the coding scheme +1 for Cr and -1 for Cg.

Supplementary Table 2: Genotype of the near-isogenic line

Scaffold	Position (nt)	Marker (see ref. 1)	Genotype of NIL SAS437<br>(a: homozygous *C. grandiflora*;<br>b: homozygous *C. rubella*;<br>h: heterozygous)
1	2299856	A05	b
1	3114855	A07	b
1	4555967	A08	b
1	6168749	A10	b
1	8858360	A12	h
1	9766939	3G33	b
1	19167208	A16	b
2	363608	CAP5	h
2	403722	B01	h
2	763403	1G63	b
2	955458	B02	b
2	1587177	1G61	b
2	7388511	B04	b
2	7816098	B06	b
2	9064596	B07	b
2	9604872	B08	b
2	12200809	B11	b
2	13270845	B10	b
3	381439	3G02	b
3	6151596	3G17	b
3	7559574	C004	b
3	13241733	C06	b
3	13513239	C10	b
4	1741932	D03	b
4	7004190	D04	b
4	7284563	D05	h
4	8632170	2G32	b

4	13451362	D08	b
5	34889	C07	h
5	1824372	E02	h
5	5274426	E05	h
5	7709629	E04	h
5	9974165	E08	h
5	13584056	CAP175	b
6	5967395	F03	h
6	9368196	F6	a
6	15009532	4G05	b
6	16513470	F12	b
6	16525594	F11	b
7	2298776	G02	b
7	4512247	G03	h
7	9972054	4G15	h
7	9569806	G07	h
7	10149356	G08	h
7	15915126	G12	b
7	16968217	G11	h
8	1078892	H03	b
8	2623520	H04	b
8	11130390	H10	b
8	13265528	H11	b

Supplementary Table 3 : List of annotated genes in the mapping intervals.

QTL	Scaffold	Start	Stop	ID	*A. thaliana* homologues	Functional annotation
QTL2	scaffold_2	202551	204786	Carubv10021457m	AT1G64300.1	Protein kinase family protein
	scaffold_2	211957	213051	Carubv10021816m	AT1G64295.1	F-box associated ubiquitination effector family protein
	scaffold_2	215677	216762	Carubv10021907m	AT1G64290.1	F-box protein-related
	scaffold_2	217298	219989	Carubv10020031m	AT1G64280.1_ NPR1	regulatory protein (NPR1)
	scaffold_2	220545	222704	Carubv10020278m	AT1G64260.1	MuDR family transposase
	scaffold_2	223775	225895	Carubv10021357m	AT1G64260.1	MuDR family transposase
	scaffold_2	227573	229999	Carubv10021783m	AT1G64260.1	MuDR family transposase
	scaffold_2	230895	233624	Carubv10020053m	AT1G64255.1	MuDR family transposase
	scaffold_2	234743	235063	Carubv10021722m	AT1G64235.1	Bifunctional inhibitor/lipid-transfer protein/seed storage 2S albumin superfamily protein
	scaffold_2	235451	237355	Carubv10020996m	AT1G64230.1_UBC28	ubiquitin-conjugating enzyme 28
	scaffold_2	237595	238024	Carubv10021245m	AT1G64220.1_TOM7-2	translocase of outer membrane 7 kDa subunit 2
	scaffold_2	238102	240015	Carubv10021395m	AT1G64210.1	Leucine-rich repeat protein kinase family protein
	scaffold_2	241053	242892	Carubv10020883m	AT1G64200.1_VHA-E3	vacuolar H+-ATPase subunit E isoform 3
QTL7	scaffold_7	9253104	9254894	Carubv10005176m	AT4G08691.1	
	scaffold_7	9254981	9258150	Carubv10005361m	AT4G17050.1_ UGLYAH	ureidoglycine aminohydrolase
	scaffold_7	9257045	9262244	Carubv10005166m	AT4G17040.1_CLPR4	CLP protease R subunit 4
	scaffold_7	9264180	9265793	Carubv10005585m	AT4G17030.1_AT-EXPR	expansin-like B1
	scaffold_7	9266354	9269322	Carubv10006310m	AT4G17020.3	transcription factor-related
	scaffold_7	9268573	9271765	Carubv10005920m	AT4G17010.1	
	scaffold_7	9272660	9275533	Carubv10006518m	AT4G17000.1	
	scaffold_7	9277237	9277809	Carubv10006767m	AT4G16980.1	arabinogalactan-protein family
	scaffold_7	9279776	9284122	Carubv10004105m	AT4G16970.1	Protein kinase superfamily protein
	scaffold_7	9291754	9296295	Carubv10004008m	AT4G16890.1_BAL,SNC1 AT4G16950.1_RPP5	disease resistance protein (TIR-NBS-LRR class), putative
	scaffold_7	9297395	9299037	Carubv10005274m	AT4G16850.1	

scaffold_7	9299185	9303240	Carubv10004893m	AT4G16845.1_VRN2	VEFS-Box of polycomb protein
scaffold_7	9303846	9304112	Carubv10007592m	AT4G16840.1	
scaffold_7	9304231	9306290	Carubv10006791m	AT4G16835.1	Tetratricopeptide repeat (TPR)-like superfamily protein
scaffold_7	9306420	9308554	Carubv10005134m	AT4G16830.1	Hyaluronan / mRNA binding family
scaffold_7	9309482	9311038	Carubv10007507m	AT4G16820.1_PLA-Ibeta2	alpha/beta-Hydrolases superfamily protein
scaffold_7	9316581	9318771	Carubv10006259m	AT4G16810.1	VEFS-Box of polycomb protein
scaffold_7	9321931	9324324	Carubv10007038m	AT4G16810.1	VEFS-Box of polycomb protein
scaffold_7	9325020	9327589	Carubv10004885m	AT4G16807.1	
scaffold_7	9328471	9329520	Carubv10007120m	AT4G16800.1	ATP-dependent caseinolytic (Clp) protease/crotonase family protein
scaffold_7	9332345	9334066	Carubv10004732m	AT4G16790.1	hydroxyproline-rich glycoprotein family protein
scaffold_7	9335010	9336719	Carubv10005275m	AT4G16780.1_ATHB-2	homeobox protein 2
scaffold_7	9345932	9348909	Carubv10005289m	AT4G16770.1	2-oxoglutarate (2OG) and Fe(II)-dependent oxygenase superfamily protein
scaffold_7	9349617	9353002	Carubv10007744m	AT4G16770.1	2-oxoglutarate (2OG) and Fe(II)-dependent oxygenase superfamily protein
scaffold_7	9353682	9356075	Carubv10006983m	AT4G16765.1	2-oxoglutarate (2OG) and Fe(II)-dependent oxygenase superfamily protein
scaffold_7	9357182	9361483	Carubv10004312m	AT4G16760.1_ACX1,ATACX1	acyl-CoA oxidase 1
scaffold_7	9364548	9365366	Carubv10005867m	AT4G16750.1	Integrase-type DNA-binding superfamily protein
scaffold_7	9374054	9375936	Carubv10007826m	AT4G16745.1	Exostosin family protein

Supplementary Table 4: **Geographic origin of the additional *C. grandiflora* populations collected during this study.**

***C. grandiflora* population**	**City, country**	**Latitude/Longitude**
P19	Kastanonas, Greece	39,84N/20.95E
Cg 926/2	Votonosi, Greece	39.76N/21.11E
P18	Vovusa, Greece	39.91N/21.05E
P11	Metsovo, Greece	39,77N/21,18E
P14	Metsovo, Greece	39.76N/21.18E
P22	Konitsa, Greece	40.05N/28.89E

Supplementary Table 5: List of the primers used in the study

Name	Usage	Position; Polymorphism *Cr*/*Cg*	Sequence (5'-3')
oAS1036	Cloning of NPR1 ORFs	n.a.	TTAATTAA ATGGACACCAATCTTGATGGATTCGC
oAS1037	Cloning of NPR1 ORFs	n.a.	TTAATTAATCACCGACGCCGATTAGAGAGTT T
oAS1137	Cloning of the 173tsRPP5	n.a.	CTCCTCTTAATTAAGTGATTTTTCTCTACAAG CGAATCTAGAGAGGATATTCGAGAAGTATTG ACCGATA
oAS1138	Cloning of the 173tsRPP5	n.a.	CTCCTCTTAATTAATTGAGACTTCCAAGTCGC AGAGTTCC
oAS1129	Genotyping of NPR1	n.a.	TTGATGCTCTTCTAGGATTTTGAAAGGTGCTA
oAS1130	Genotyping of NPR1	n.a.	GACTCGGATGATATCACGCTAGTCAATTTGC
oAS873	Genotyping CrRPP5	n.a.	CAAACCATAATAGTAAGATGCTTCAGTTACC AG
oAS1123	Genotyping CrRPP5	n.a.	TCGAATACCTCGATCTCAGTGGTT
oAS839	genotyping CgRPP5	n.a.	GCTGTTGATGCTTAAGGAGGTTCCC
oAS1133	genotyping CgRPP5	n.a.	GTTAATGATGGGAAGAACACCTTTTAC
oAS1276	genotyping CgRPP5	n.a.	ATGAAAATCTCCACTCAAGTAGTGTCCACAC
oAS1072	qPCR *CaNPR1*	n.a.	CACCGAGTCCTCTATCGTTTATC
oAS1073	qPCR *CaNPR1*	n.a.	GTCAGCGAGAACGAGCTTAG
oAS1084	qPCR *caTUB6*	n.a.	GCTCCTTCAGTGTAGTGCCC
oAS1085	qPCR *caTUB6*	n.a.	CAGAACTGGCCCTTATGGTC
oAS1068	qPCR *CaRPP5*	n.a.	GACGATGAGATTAAAATGACTTCTGCCA
oAS1069	qPCR *CaRPP5*	n.a.	AGCGCCTTGAGAAGATGACTAAGGAA
oAS1145	qPCR *Carubv10025432m*	n.a.	GTAACCTGTACCAATTTGTACCCATC
oAS1146	qPCR *Carubv10025432m*	n.a.	TGTAGAGATGCCATCCTGGCG
oAS1074	qPCR *CaPR1*	n.a.	TTGCAACTGATGATGGTTCC
oAS1075	qPCR *CaPR1*	n.a.	TAGTGGCGACTTGTCTGGTG
oAS1111	qPCR *CaEDS5*	n.a.	TCTTTATTGGTTTGGTTTGTGG
oAS1112	qPCR *CaEDS5*	n.a.	CAGTAACAGCCCAAGGTCC
oAS1113	qPCR *CaEDS1*	n.a.	TCGGAAGGAGAAATACACGG
oAS1114	qPCR *CaEDS1*	n.a.	GCTCTTACCGGAATCAATGG
oAS1078	qPCR *CaPDF1.2*	n.a.	TTATGCGAGAGGTCAAGTGG
oAS1079	qPCR *CaPDF1.2*	n.a.	GATCCATGTCGTGCTCCTTC
oAS1107	qPCR *PR2*	n.a.	TCTCAGACACCCCGATCTTC
oAS1108	qPCR *PR2*	n.a.	CCACATGTATAACTCGGGCC
oAS1109	qPCR *CaPAD4*	n.a.	AAGAAAGGCAGCACATAGATCC
oAS1110	qPCR *CaPAD4*	n.a.	ACAAAGCTCGCGAAGAGAAG
oAS1080	qPCR *CaORA59*	n.a.	ACCGCCCTAGAGGAAAGAAG
oAS1081	qPCR *CaORA59*	n.a.	TCACTTTCTTGCGTCGTGAC
oAS928	HiB1 CAPS markers using *HindII*	scaffold_2:75028; C/T	GGTTCGTCGGCGCACACATTGAG
oAS929	HiB1 CAPS markers using *HindII*		CTCACTTCTCAATCTCTCTCGGTAATG

oAS926	HiB2 dCAPS markers using *ClaI*	scaffold_2:129775; C/T	TCGAACGGCAATCTTCAAGTTCATCGA
oAS927	HiB2 dCAPS markers using *ClaI*		CTATTCTCGACGACGAGCTTCTAAG
oAS1001	HiB3 CAPS markers using *NlaIII*	scaffold_2:203131 ; A/G	AGTCAGAAGCAGGACCAGAGAGTTGTC
oAS1002	HiB3 CAPS markers using *NlaIII*		CACACCTGATCCTATAATCTGGTATGCAC
oAS1039	HiB4 CAPS markers using *DdeI*	scaffold_2:241249 ; G/A	AACCAGATCCACCAGATGGTCTGCT
oAS1040	HiB4 CAPS markers using *DdeI*		AAGATCAAGATCAAGAGAGAGAGAAGAATT
oAS991	HiB5 CAPS markers using *ApoI*	scaffold_2:249356 ; C/T	CGGTTCAGTACTTTAAGAAGTTCGGTT
oAS992	HiB5 CAPS markers using *ApoI*		GTTCTGGAATTTCCCAATTGCTCTG
oAS917	HiB6 CAPS markers using *AluI*	scaffold_2:252505 ; G/C	TAGTGACATTGACATAGAAGAGTGATGGG
oAS918	HiB6 CAPS markers using *AluI*		GTGTGACTCTAGCATAAGGTATTGTAAGCTC
oAS916	HiB7 dCAPS markers using *DdeI*	scaffold_2:303580 ; G/A	ACAAGTGGGCAGACAAGCCACTCA
oAS910	HiB7 dCAPS markers using *DdeI*		CTTACCGTATCATTTTCAAGAACATGACAGA
CAP5_F	HiB8 dCAPS markers using *PstI*	scaffold_2:363608 ; G/T	TCACCTCTTTGGTCACACTGCA
CAP5_R	HiB8 dCAPS markers using *PstI*		TGGATTGCGTGATTTTGTT
oAS797	HiG1 CAPS markers using *TaqI*	scaffold_7:8868399 ; G/A	CTCAATGGTGCAGTTACATCAAACTCTC
oAS798	HiG1 CAPS markers using *TaqI*		AATGTGGTGCATTTAGGCAACAGC
oAS808	HiG2 dCAPS markers using *DdeI*	scaffold_7:9135785 ; A/G	ACAAGAATGGGGACAATGGATAACC
oAS809	HiG2 dCAPS markers using *DdeI*		GAATCGTGATATCGTATCTCTCATTTG
oAS810	HiG3 dCAPS markers using *XcmI*	scaffold_7:9106757 ; A/G	AGCTGGAGAACAGTTCTGTGTCACT
oAS811	HiG3 dCAPS markers using *XcmI*		TTTTTGGAATTGCATCCATGGAAGTAT
oAS1249	HiG4 Indel markers	scaffold_7.9253895; GAAAGGGAGTTAGGAG/TAAGCTAAACTCCAGGGTTCGANNNNNNNNNNTGTTTTACTTGGGTGCAGTTGG	AAGCTAAACTCCAGGGTTCGA
oAS1250	HiG4 Indel markers		TGTTTTACTTGGGTGCAGTTGG
oAS806	HiG5 CAPS markers using *XmnI*	scaffold_7:9374725 ; A/C	CGTTGCAGAGAGCTTTAATAGAATTTC
oAS807	HiG5 CAPS markers using *XmnI*		GTCGCTTGCCACGATTGGGTAATA
G07_F	HiG6 CAPS markers using *HinfI*	scaffold_7:9569807 ; C/T	AAGGATGCTGTCAGCTGCCTTCTTG
G07_R	HiG6 CAPS markers using *HinfI*		TGGATACAGGTGGCCTATGGTGTC
n.a. not applicable			

Supplementary Discussion

Genetic incompatibilities in the genus *Capsella*.

The genetic incompatibility in the *Cr1504* x *Cg926* RIL population suggested that the presence within the same cells of RPP5 and $NPR1^{go}$ induces an autoimmune response. If true, any crosses combining a functional RPP5 protein with $NPR1^{go}$ should recreate the stunted growth phenotype. We tested this hypothesis by performing random crosses between seven *C. rubella* and nine *C. grandiflora* accessions (Supplementary Fig. 7). A phenotype similar to the *Cr1504* x *Cg926* incompatible hybrids was segregating in 13 of the resulting F_2 populations.

The *RPP5* alleles from three of the *C. rubella* accessions used for the crosses (*Cr4.23*, *Cr86IT1-C* and *Cr1377/5*) belong to different haplotype groups than *Cr1504RPP5* (Supplementary Fig. 5b), while no *RPP5* could be detected in a fourth *C. rubella* accession (*Cr 1GR1-TS1*). Among the *C. grandiflora* x *C. rubella* F_2 progenies descending from these *C. rubella* accessions, we did not observe any correlation between the *RPP5* genotype (as determined by a tightly linked molecular marker HiG2 to be able to assess the genotype at the *RPP5* locus also in progeny of *Cr 1GR1-TS1*) and the stunted growth. Similarly, no correlation was observed in the F_2 population descending from *Cr22.5* in cross 21, whose *RPP5* allele belongs to the same haplotype group as *Cr1504RPP5*. We note that the absence of incompatible phenotypes in cross 12, also involving *Cr22.5*, and in cross 4 is not informative, as the *C. grandiflora* parental plants of these crosses did not pass on an $NPR1^{go}$ allele to the respective F1 plants. This is due to the *C. grandiflora* accession *Cg926* being heterozygous for $NPR1^{go}/NPR1^{rub}$ (see also Fig. 3a). In fact, a robust correlation between incompatible phenotype and *RPP5* genotype was only seen in F_2s descending from crosses between *Cr1504* and diverse *C. grandiflora* individuals. These results indicate that only a specific *RPP5* haplotype is incompatible with the $NPR1^{go}$ alleles. It is therefore likely that after the divergence of *C. rubella* from *C. grandiflora*, a novel mutation in *Cr1504RPP5* has rendered it incompatible with $NPR1^{go}$. This hypothesis is supported by the observation that no correlation between stunted growth phenotypes and *RPP5/NPR1* genotypes was observed within natural *C. grandiflora* populations.

In addition, modifier alleles of the *NPR1/RPP5* incompatibility appear to be segregating in *C. grandiflora*. This is based on the observation that the strength and frequency of the stunted growth varies between the different F_2s descending from *Cr1504*. For example, although the same $NPR1^{go}$ and *RPP5* haplotype are segregating in the crosses 6 and 7, only 10 % of the

F_2s show an incompatible phenotype in cross 7 versus 30% in cross 6 (Supplementary Fig. 6A,S7), and the phenotypic severity is reduced in the cross 7 relative to the cross 6 F_2 progenies (Supplementary Fig. 7B). Since *Cr1504* is highly inbred, this indicates that modifiers affecting the penetrance and expressivity of the genetic incompatibility are segregating within *C. grandiflora*.

Importantly, we observed that $NPR1^{go}$ haplotypes are also associated with genetic incompatibilities that do not involve *RPP5* (Supplementary Fig. 7; crosses 3 and 9). In the progeny of cross 9, all plants homozygous for the $NPR1^{go}$ allele showed the incompatible phenotype, while none of the plants homozygous for $NPR1^{rub}$ did so, yet there was no difference in the *RPP5* genotype frequency between plants with incompatible and compatible phenotypes. Similarly, in the F_2 of cross 3, in which no *RPP5* presence could be detected, all but one of the 39 incompatible hybrids had at least one copy of the $NPR1^{go}$ allele, and $NPR1^{go}$ homozygotes were underrepresented amongst compatible plants. Individual $NPR1^{rub}$ homozygotes amongst the incompatible plants were also observed in two of the crosses involving *Cr1504*, which is likely due to mis-scoring of the phenotype or to unrelated deleterious mutations from the *C. grandiflora* parent that became homozygous in the F_2. Given also the molecular evidence for a constitutive auto-immune response in the incompatible plants in the F_2 from cross 3 (Supplementary Fig. 8a,b), we consider it highly likely that this incompatibility is also caused by the $NPR1^{go}$ allele. In the F_2 of cross 10, the stunted growth phenotype co-segregated with the $NPR1^{rub}$ allele, but not with the *RPP5* genotype (Supplementary Fig. 7). While suggestive of an involvement of $NPR1^{rub}$ in the incompatibility, several incompatible plants were homozygous for $NPR1^{go}$, and further experiments will be needed to test a causal role for $NPR1^{rub}$. In any case, these results indicate that alleles at additional loci are incompatible with $NPR1^{go}$ and possibly $NPR1^{rub}$; these alleles are likely to segregate in *C. grandiflora*, as the incompatibilities were only observed in some, but not all crosses involving a given *C. rubella* accession (compare for example crosses 3, 20 and 24). Together, these results support the notion that the two *NPR1* haplotype groups with their strong sequence divergence resulting from long-term balancing selection facilitate the establishment of genetic incompatibilities by mutations to interacting loci. If any such strongly divergent haplotypes were sorted into derived populations in a mutually exclusive manner, for example following genetic bottlenecks such as during independent transitions to selfing from an ancestral outbreeding species, the first step to the formation of a gene-flow barrier would already have been achieved, and only one further

mutation to an interacting locus in one of the derived populations would be required for the establishment of a BDMI.

Supplementary Reference

1. Sicard, A. *et al.* Genetics, evolution, and adaptive significance of the selfing syndrome in the genus Capsella. *Plant Cell* **23**, 3156-3171 (2011).
